# The effect of whole blood logistics on neutrophil non-specific activation and kinetics ex vivo

**DOI:** 10.1038/s41598-023-50813-x

**Published:** 2024-01-30

**Authors:** Chao Li, Mehtab Farooqui, Ravi Chandra Yada, Joseph B. Cai, Anna Huttenlocher, David J. Beebe

**Affiliations:** 1 Carbone Cancer Center, University of Wisconsin-Madison, Madison, WI USA; 2 Department of Biomedical Engineering, University of Wisconsin-Madison, Madison, WI USA; 3 Department of Pathology and Laboratory Medicine, University of Wisconsin-Madison, Madison, WI USA; 4 Department of Cellular and Molecular Biology, University of Wisconsin-Madison, Madison, WI USA; 5 Department of Medical Microbiology and Immunology, University of Wisconsin-Madison, Madison, WI USA; 6 Department of Pediatrics, University of Wisconsin-Madison, Madison, WI USA

**Keywords:** Cell death and immune response, Immune cell isolation

## Abstract

While the exquisite sensitivity of neutrophils enables their rapid response to infection in vivo; this same sensitivity complicates the ex vivo study of neutrophils. Handling of neutrophils ex vivo is fraught with unwanted heterogeneity and alterations that can diminish the reproducibility of assays and limit what biological conclusions can be drawn. There is a need to better understand the influence of ex vivo procedures on neutrophil behavior to guide improved protocols for ex vivo neutrophil assessment to improve inter/intra-experimental variability. Here, we investigate how whole blood logistics (i.e., the procedure taken from whole blood collection to delivery of the samples to analytical labs and storage before neutrophil interrogation) affects neutrophil non-specific activation (i.e., baseline apoptosis and NETosis) and kinetics (i.e., activation over time). All the experiments (60+ whole blood neutrophil isolations across 36 blood donors) are performed by a single operator with optimized isolation and culture conditions, and automated image analysis, which together increase rigor and consistency. Our results reveal: (i) Short-term storage (< 8 h) of whole blood does not significantly affect neutrophil kinetics in subsequent two-dimensional (2D) cell culture; (ii) Neutrophils from long-term storage (> 24 h) in whole blood show significantly higher stability (i.e., less non-specific activation) compared to the control group with the isolated cells in 2D culture. (iii) Neutrophils have greater non-specific activation and accelerated kinetic profiles when stored in whole blood beyond 48 h.

## Introduction

Neutrophils, the most abundant immune cells in our body, form the first line of defense in human immunity. While recently increased efforts have shifted towards studying the multifaceted role of neutrophils in many diseases, e.g., cancer and infections^1,2^, there is still a call for further understanding the basics of neutrophil kinetics including survival (or cell death), activation, and migration both in vivo and ex vivo^3^. For example, the commonly cited “short lifespan of neutrophils” thought to be 7–9 h is actually the disappearance rate of neutrophils in the blood^4^, which does not necessarily account for the lifespan of neutrophils in different compartments (e.g., bone marrow, tissue)^3^. Previous studies have suggested a lifespan of neutrophils for up to 3–5 days in vivo^5^. Although this work has been met with criticism and challenged by other groups, there is interest in investigating the potential time scale in which neutrophils are still viable^6,7^. When neutrophils are exposed to external insults (e.g., tissue injury) and/or invading substances, the innate immune cells can become activated and intervene through phagocytosis (i.e., ingestion of pathogens), secretion of antimicrobials via degranulation, and a newer third function—NETosis [formation and release of neutrophil extracellular traps (NETs)]^8^. The many potential triggers, the activation pathways, and the emerging functions of NETosis (e.g., suicidal NETosis versus vital NETosis) are still under investigation^9,10^.

Human immunity acquired critical plasticity and adaptability from exposure to constantly changing local challenges and conditions. Recent studies have shown that the environment plays a greater role in shaping the immune system compared to most heritable factors^11,12^. These insights underscore the importance of better understanding the interplay between environmental conditions and immune response. Furthermore, other groups have shown that whole blood collection method and anticoagulants, the storage temperature of neutrophils after isolation^13^, autologous signaling molecules^14^, oxygen tension in the culture environment^15,16^, and co-culture conditions^17^ are all important factors to consider when assaying neutrophil behavior ex vivo (see summary in Table 1). Baseline neutrophil characteristics, including activation state, kinetic profiles, and functional outputs [e.g., reactive oxygen species (ROS) production, cytokine secretion] are influenced by a multitude of tunable external factors and can quickly shift toward non-physiological conditions. Overall, these findings suggest that neutrophils are sensitive to their culture environment, and optimizing these parameters can be helpful to make studies more rigorous and reproducible. However, these studies were limited by inconsistent isolation methods, variable culture conditions (e.g., media composition, culture times, cell stains), and multiple operators which can all lead to confounding variables that make downstream analysis inconsistent and parallel comparison of the conclusions (from different labs) difficult. During the in vivo to ex vivo transition neutrophils are subjected to a multitude of altered environmental conditions; therefore, baseline neutrophil kinetics are already affected and potentially skewed before and during interrogation in an assay, so identifying optimized cell culture and storage parameters is essential for generating robust and reliable data. These variations are further amplified when working with whole blood samples collected in the clinical setting when the time from blood collection to neutrophil interrogation (i.e., whole blood logistics) can range from hours to days depending on the final entity/institution assaying for molecular and functional readouts. Establishing a standard set of practices for whole blood handling and culture/interrogation of neutrophils after blood collection can make results more comparable across experimental replicates, between different labs, and minimize unwanted heterogeneity.

**Table 1:** Recent studies of human neutrophil kinetics in ex vivo cell culture environments.

Isolation method	Cell culture condition	Donor	Factors investigated	Conclusion(s)*
Gradient neutrophil isolation with red blood cell (RBC) lysis (Percoll)	2D monoculture with a water bath incubation (37 °C, air, i.e., 21% oxygen [O_2_], 0.04% carbon dioxide [CO_2_]), RPMI medium, up to 7 h	Healthy	Blood collection techniques, anticoagulants, storage temperature of isolated neutrophils	(Krabbe et al*.*)^11^ Minor effects of these factors on the isolation of neutrophils were observed, which helps reduce the parameter space in a study. This study also suggested a relatively short (1 h) storage window between isolation and interrogation while maintaining an optimal storage temperature of 37 °C to stabilize the isolated neutrophils
Gradient neutrophil isolation with RBC sedimentation or lysis (Percoll and Ficoll)	2D monoculture with a standard CO_2_ incubator (37 °C, 21% O_2_, 5% CO_2_, 95% relative humidity [RH]), RPMI medium + 10% autologous plasma, up to 60 h	Healthy	Autologous plasma (10% supplement) versus fetal bovine serum (FBS)	(Alipour et al*.*)^12^ Neutrophils cultured in autologous plasma showed reduced apoptosis
Gradient neutrophil isolation with RBC sedimentation (Percoll)	2D monoculture with a hypoxia incubator (37 °C, 3% O_2_, 5% CO_2_, 95% RH), Iscove′s Modified Dulbecco′s Medium (IMDM) + 10% autologous serum, up to 20 h	Healthy	Hypoxia (3% O_2_)	(Walmsley et al*.*)^13^ Hypoxia increased neutrophil survival by inhibiting apoptosis mediated by HIF-1α–dependent NF-κB activity
Gradient neutrophil isolation (Percoll)	2D monoculture with a hypoxia incubator (37 °C, 21% to 1% O_2_, 5% CO_2_, 95% RH), IMDM + 10% autologous serum, up to 96 h	Healthy	Hypoxia (1% O_2_)	(Marwick et al*.*)^14^ GM-CSF and inflammatory conditioned media increased neutrophil survival under hypoxia (1% O_2_) with cells staying functionally active even at 96 h
Negative selection-based neutrophil isolation with RBC lysis (MACSxpress)	A microscale 3D co-culture model with a standard CO_2_ incubator, iCell-endothelial cells (iECs) medium, up to 24 h	Healthy	Interaction between blood endothelial cells and neutrophils in a bacterial infection	(Hind et al*.*)^15^ Co-culture with blood endothelial cells increased neutrophil lifespan (via GM-CSF) and migration (via IL-6) in the inflammatory environment

*Different isolation methods and culture conditions (including incubator, culture media, oxygen level, 2D vs 3D, culture time, and cell stains) are adopted from lab to lab, which makes the results difficult to be compared in parallel and to reflect the complete kinetic profiles of neutrophils (i.e., the lifespan of neutrophils in vivo is still under scrutiny)^5–7^.

Here, we seek to reduce non-specific neutrophil activation (i.e., the baseline apoptosis and NETosis) and maintain their standard kinetic profiles (i.e., how the cells get activated over time in a specific ex vivo environment) at baseline (i.e., immediately after isolation, prior to interrogation) while trying to understand the external parameters that influence these characteristics. To this end, we identified and applied optimal neutrophil isolation strategies and 2D culture environment conditions (e.g., oil overlay, incubator system, length of culture, cell staining concentrations, and seeding densities) while investigating more downstream variables in neutrophil interrogation. We also investigate how disparities in the time between whole blood collection and neutrophil isolation prior to interrogation can contribute to baseline properties (e.g., non-specific activation and kinetics) of isolated neutrophils. Additionally, to the best of our knowledge, no previous work has studied how long-term (up to 72 h) neutrophil storage in whole blood can influence neutrophil behavior. This unique parameter is important to further elucidate since neutrophils are not always immediately isolated due to technical or logistical limitations (e.g., clinical settings or distant site of end point analysis). Through this work we highlight how neutrophil storage in whole blood at physiologic temperature (37 °C) across multiple days can minimize non-specific activation observed in time-matched 2D culture, while exhibiting accelerated kinetic profiles relative to baseline. All the wet lab work was completed by a single operator to minimize potential complications of operator-to-operator heterogeneity to increase the robustness of this study. Additionally, measurements were performed by an automated analysis pipeline to further reduce bias and increase the throughput of this work. This study was performed using 60 + whole blood samples across 36 unique donors. The neutrophil isolation was completed by a single operator to clarify parameters for ex vivo whole blood handling and established a framework for exploring other modifiable external factors that can affect neutrophil behavior.

## Methods

### Whole blood collection

All blood samples were drawn according to Institutional Review Boards (IRB) approved protocols per the Declaration of Helsinki at the University of Wisconsin-Madison in the Beebe Lab (IRB# 2020-1623, healthy donors) and in the Lang Lab (IRB# 2014-1214, cancer patients). Informed consent was obtained from all subjects in the study. Whole blood was collected with standard ethylenediaminetetraacetic acid (EDTA) tubes (BD, #36643, EDTA [K2] STERILE, 1054688) and then stored at RT (~ 22 °C) or 37 °C in stationary storage or rotary storage (Thermo Scientific, Labquake, Tube Rotator, 415110Q, 6 rpm) before isolation.

### Neutrophil isolation

Neutrophils were isolated from whole blood using magnetic bead-based negative selection. Two negative selection kits—MACSxpress Whole Blood Neutrophil Isolation Kit, human (Miltenyi Biotec, 130-104-434) and EasySep Direct Human Neutrophil Isolation Kit (STEMCELL, 19666)—were compared. In MACSxpress isolation, RBCs were further depleted from the neutrophil pellet using BD Pharm Lyse buffer (BD Biosciences, 555899), according to the manufacturer’s instruction. In EasySep isolation, the neutrophil pellet was directly resuspended in appropriate media for further experiments. Cell count of each isolation was obtained using a hemocytometer (LW Scientific).

### 2D cell culture ex vivo

The cell culture was performed in standard 384-well plates (Corning Clear Flat Bottom Polystyrene Tissue Culture-treated Microplates, 3701) in a standard CO_2_ incubator (37 °C, 21% O_2_, 5% CO_2_, 95% RH) (Thermo Scientific, HERACELL 240i), or an onstage incubator (37 °C, 21% to 0% O_2_, 5% CO_2_, 95% RH) (Bold Line, Okolab). After isolated from whole blood, neutrophils were resuspended in culture media and then seeded into each well at a seeding density ranging from 1 × 10^4^ to 2 × 10^5^ cells per well in 20 µl culture media with and without oil overlay [Fluorinert FC-40 (abbreviation “FC40”), 10 µl per well, Sigma Aldrich, F9755; silicone oil, 50 µl per well, Sigma Aldrich, 317667 (5 cSt, abbreviation “SO5”), 378364 (100 cSt, abbreviation “SO100”)]. Neutrophils were cultured in RPMI (Gibco) + 10% FBS + 1% P/S unless otherwise specified.

### Microscopic imaging

Bright-field, fluorescence images and videos were recorded on a Nikon Ti Eclipse inverted epifluorescence microscope (Nikon Instruments). The culture plate was kept at 37 °C, 21% or 5% O_2_, 5% CO_2_, and 95% RH in an onstage incubator (Bold Line, Okolab) during imaging or time lapse. Cells were stained with Hoechst (ThermoFisher Scientific, 62249), Sytox (ThermoFisher Scientific, S7020), and Annexin V (ThermoFisher Scientific, A13203) at the desired concentrations as described above.

### Nucleus circularity analysis

The fluorescence images of × Hoechst (30 magnification) were batch processed and analyzed using a customized Java code (Github—https://github.com/jcai0791/Neutrophil-Project) calling functions from Fiji ImageJ (Supplementary Fig. 2). The workflow includes: (1) Open Fiji ImageJ. (2) Analyze → Set Measurements… (select “Area”, “Perimeter”, and “Shape descriptors”). (3) Open a Hoechst image (Image → Type → 16-bit). (4) Process → Subtract Background… (Rolling ball radius: 50 pixels). (5) File → Save As → Jpeg… (use default file name). (6) Image → Adjust → Threshold… (pick a Method, e.g., Minimum, select “Dark background”). (7) File → Save As → Jpeg… (use default file name + Threshold- “Method, e.g., Minimum”). (8) Analyze → Analyze Particles… [Size (μm^2^): 0-Infinity, Circularity (0.00–1.00), Show: Bare Outlines]. (9) File → Save As → Jpeg… (use default file name + Outlines). 10) Save Results (click the “x” button and select “Save”, use default file name.csv).

### Area fraction analysis

The fluorescence images of Sytox and Annexin V (× 4 magnification) were batch processed and analyzed using a customized Java code (Github—https://github.com/jcai0791/Neutrophil-Project) calling functions from Fiji ImageJ (Supplementary Fig. 3). The workflow includes: (1) Open Fiji ImageJ. (2) Analyze → Set Measurements… (select “Area fraction”). (3) Open a set of the original .nd2 images with Sytox and Annexin V channels (Image → Type → 16-bit). (4) Image → Duplicate… (Separate the Sytox and Annexin V channels by making a duplicate). (5) Process → Subtract Background… (Rolling ball radius: 50 pixels). (6) Image → Adjust → Threshold… (pick a Threshold Method, Default for the Sytox channels, or MaxEntropy for Annexin V channels, select “Dark background”). (7) Analyze → Measure. (8) File → Save As → Jpeg… (use “default file name + Threshold Method-Sytox or Annexin V”). (9) Save Results (File → Save as… “file name.csv”). The data points in the .csv files were organized in the order of time points with conditions under each time point from the group of Sytox or Annexin V channels. (10) To plot the data points, we sorted the data points in the order of conditions with time points under each condition from the group of Sytox or Annexin V channels. (11) The sorted data were plotted in GraphPad Prism 9.3.0.

### Statistical analysis

Raw data were directly used in statistical analysis with no data excluded. Data were averaged from at least 3 replicates (unless otherwise stated) and present as mean ± s.d. if applicable. The statistical significance was specified in the figure captions. All statistical analyses were performed using GraphPad Prism 9.3.0.

## Results

### Neutrophil stages and the identification method

**Figure 1:**
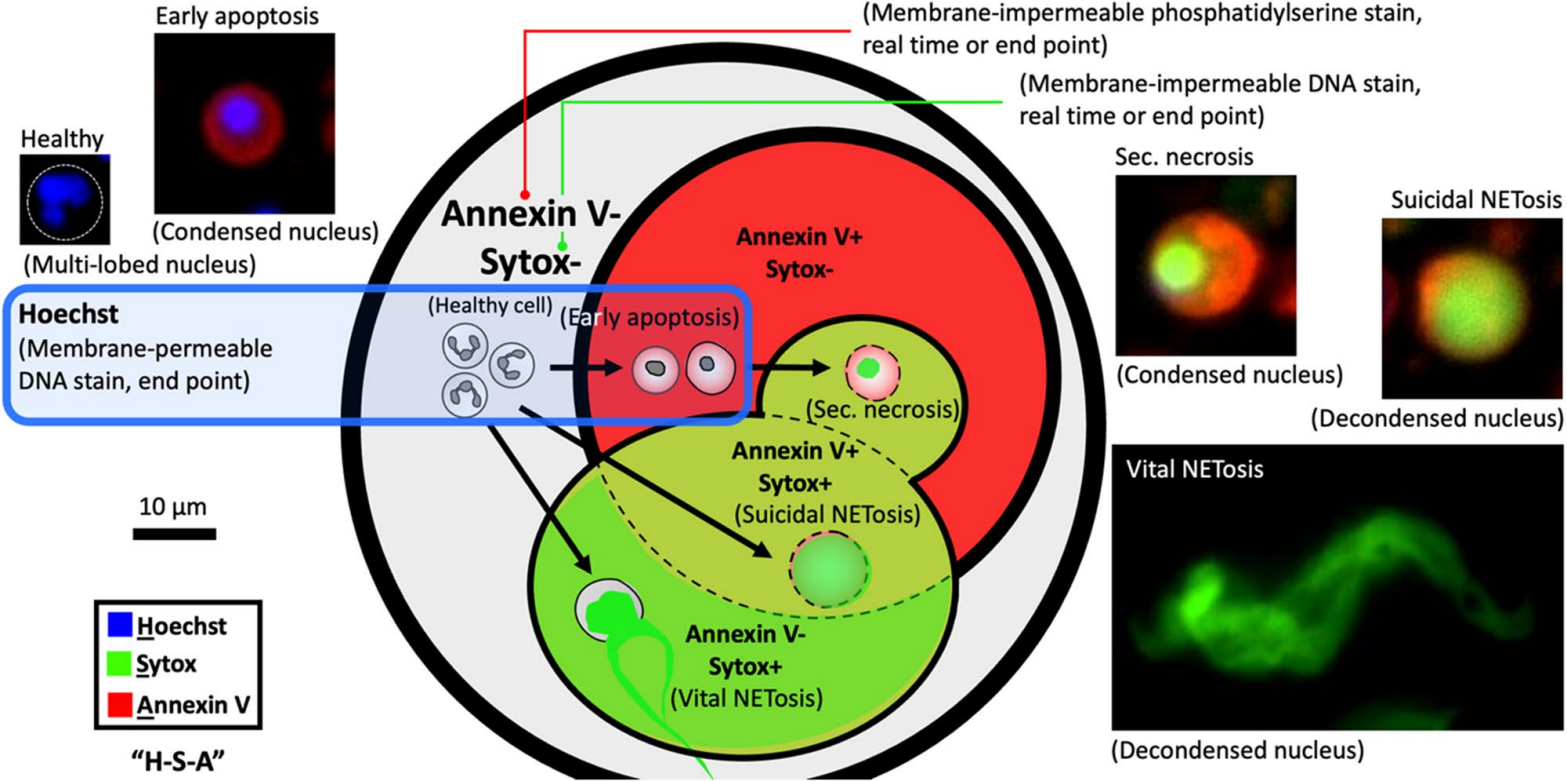
Neutrophil stages and staining readouts. A Venn diagram showing the three-dye—Hoechst-Sytox-Annexin V (H–S-A)—method (see detail in Table 2, Supplementary Fig. 1). The cell stages that Hoechst stains in H–S-A are highlighted by the blue-line box. The cell stages that Sytox stains in H-S-A are highlighted by bright green + olive green. The cell stages that Annexin V stains in H-S-A are highlighted by red + olive green. The inset microscopic images show the typical morphology/stains of neutrophil in different stages including healthy cell, early apoptosis, secondary necrosis, suicidal NETosis, and vital NETosis.

Mature, healthy (non-activated) neutrophils take a unique multilobed-shaped nucleus, which gives the cells high malleability and motility^18^. When exposed to tissue injury or infection in vivo, neutrophils are activated, responding to the lesion in different types of cell death (Fig. 1)—primarily apoptosis and NETosis^9,19^. Based on the mechanism of cell death, the shape of the neutrophil nuclei is distinguishably different. During apoptosis the chromatin condenses while during NETosis the chromatin decondenses^20^. Apoptosis and NETosis can be distinguished by analyzing the shape of nuclei with a membrane-permeable DNA stain (Hoechst)^21^. NETosis is known to have two distinct subtypes—suicidal NETosis (causing cell death) and vital NETosis (no immediate cell death)^9^. Suicidal NETosis features ruptured cytoplasmic membrane and release of diffused NETs. By contrast, vital NETosis features intact cytoplasmic membrane and release of stringy NETs. Release of NETs can be visualized by membrane-impermeable DNA stains (e.g., Sytox or propidium iodide)^21^. The rupture of cytoplasma membrane (in apoptosis and suicidal NETosis) can be visualized by phosphatidylserine stain (Annexin V)^20^.

**Table 2:** Summary of the H-S-A three-dye method for neutrophil stage readouts.

Cell stain	Molecular function	Cellular component	Neutrophil stage	Note (Supplementary Fig. 1)	Readout
Hoechst	Membrane permeable DNA stain	Nuclei	Healthy cells Early apoptosis	Hoechst is only used for end-point staining to avoid DNA damage and artifacts Hoechst doesn’t stain the DNA content in the presence of Sytox Hoechst shows poor staining contrast in human blood plasma	Circularity of nuclei (Supplementary Fig. 2)
Sytox	Membrane impermeable DNA stain	NETs (i.e., decondensed nuclei) Condensed nuclei (with ruptured cytoplasm membrane)	Suicidal NETosis Vital NETosis Sec. necrosis	Sytox can be used for either real-time or end-point staining Real-time staining causes positive error, i.e., more non-specific activation of neutrophils, due to cytotoxicity especially when used at high concentration compared to no-dye control End-point staining causes negative error due to the dissociation of NETs during pipet mixing	Area fraction (Supplementary Fig. 3)
Annexin V	Membrane impermeable phosphatidylserine stain	Cytoplasm membrane (of dying and dead cells)	Early apoptosis Sec. necrosis Suicidal NETosis	Annexin V can be used for either real-time or end-point staining Annexin V doesn’t stain when used in human plasma or RPMI supplemented with human plasma but functions normally in HPLM supplemented with human plasma (Supplementary Fig. 1)	Area fraction

In this work, we adopted Hoechst-Sytox-Annexin V (H-S-A) as a three-dye method. As summarized in Table 1, (i) Hoechst is a DNA stain that penetrates intact plasma/nuclear membranes. Hoechst stains DNA of neutrophils during all stages, including healthy cells, early apoptosis, sec. necrosis, suicidal NETosis, and vital NETosis. It’s worth noting that Hoechst shows little staining on NETs (decondensed chromatin) in the presence of Sytox (Thermo Fisher Scientific), likely due to the competition between the two dyes and the dissociated structure of the chromatin. Hoescht staining is normal on condensed nuclei in sec. necrosis when co-stained with Sytox. Hoechst intercalates into the double strands of DNA in living cells, which can affect aspects of cell function including cell division. To this end, Hoechst was only used for end-point staining (Supplementary Fig. 1). (ii) Sytox Green is a DNA stain that is impermeable to intact plasma/nuclear membranes. When the plasma/nuclear membrane is compromised/ruptured, Sytox stains any DNA content exposed to the dye. Sytox stains the condensed chromatin of neutrophils in sec. necrosis, and NETs from neutrophils that undergo NETosis. (iii) Annexin V (Thermo Fisher Scientific) stains phosphatidylserine (or the “Eat Me” molecules) on the plasma membrane of dying or dead cells and thus it stains neutrophils in early apoptosis, sec. necrosis, and suicidal NETosis. Sytox and Annexin V were used for real-time staining (i.e., the dye was added to the cell stock before cell seeding and present through a culture) to monitor neutrophil kinetics in culture (Supplementary Fig. 1).

Next, we optimized the H-S-A staining protocol to minimize the potential effects that fluorescence labeling has on normal cell function^22^. Here, we tested three different concentrations of the Sytox dye and the influence of Sytox and Annexin V on each other (Supplementary Fig. 4). For Sytox, 10,000× and 5000× dilution made no significant difference on non-specific activation and neutrophil kinetics compared to the no-dye control (Supplementary Fig. 5). Sytox at 1000× dilution caused higher activation especially after 6 h from the initiation of cell culture (Supplementary Fig. 4a,b). The combined use of Annexin V (20× dilution) with Sytox (5000× dilution) led to an increased Sytox signal but interestingly, there was no difference in the Annexin V signal compared to the Annexin V only control group (Supplementary Fig. 4c,d). To minimize the artifact from real-time staining (Supplementary Fig. 1a,b), we chose to use 10,000× Sytox with 40× Annexin V. Hoechst was only used for end-point staining to avoid any artifacts in cell function and prevent DNA damage^23^.

### Isolation method

Different isolation methods have been developed and used for isolation of primary neutrophils from whole blood (e.g., gradient neutrophil isolation with RBC sedimentation or lysis, negative selection-based neutrophil isolation with or without RBC lysis). Previous work has shown that different isolation methods can affect neutrophil phenotype and activity in functional assays; therefore, we explored two commonly used methods to identify a method best suited for this study^24^. Here, we compared two negative selection kits [MACSxpress (with RBC lysis) versus EasySep (without RBC lysis) following the manufacturer’s instructions] (Supplementary Fig. 6a). The results showed that the non-specific activation right after isolation was comparable between the two kits but noticeably MACSxpress based isolation led to accelerated kinetics in 2D culture of the neutrophils (Supplementary Fig. 6b,c). Therefore, we chose to use EasySep negative selection without RBC lysis in this work.

### House-keeping factors in 2D culture of neutrophils

Different research groups may use a range of culture conditions in their studies, which likely introduces variability and impedes reproducibility/comparability of the results between studies. Here we investigate how the selection of culture conditions may influence non-specific activation and neutrophil kinetics in standard 2D monoculture ex vivo. Based on our previous experience with microscale cultures, we recognized the importance that media evaporation, incubator system, and seeding density may all play key roles in maintaining proper cell behavior.

Microscale cell culture methods possess many advantages over bulk-scale (e.g., petri dish, culture flask) methods due to their ability to better recapitulate the parameters of in vivo cellular microenvironments (e.g., structural hierarchy, fluidic dynamics, and mass transport). Due to the small volume (< 100 μl), media loss via evaporation becomes a significant variable that exerts stress to the cells in culture. Recently, under-oil open microfluidic systems (UOMS) were developed and introduced to microscale cell culture^25–29^. UOMS enables several unique functions in microscale cell culture including (i) free physical access to the culture system with minimized evaporation and sample contamination^25,26,30^, (ii) versatile open fluidic controls^27^, and (iii) autonomously regulated oxygen microenvironments (AROM)^29^. We tested the isolated neutrophils in standard 2D monoculture from nine donors with and without oil overlay (Supplementary Fig. 7a). In five of the nine randomly selected donors, oil (especially silicone oil) overlay led to less non-specific activation compared to the no-oil culture, as measured by the area fraction of the Sytox signal at the end of the stationary phase (Supplementary Fig. 7b, see the definition in Fig. 3a). For the remaining four donors, silicone oil overlay did not significantly affect the activation status of the neutrophils. There was no obvious pattern in which donor neutrophils would benefit from the silicone oil overlay. Therefore, for subsequent experiments we used a silicone oil (coded in tan color in the figures) overlay in 2D culture to get improved culture environment stability and consistency.

Next, we compared two incubator systems—a standard incubator (SI) versus an onstage incubator (OI) (Supplementary Fig. 8a). They all allow the operators to select and control the O_2_ level, CO_2_ level, temperature, and RH. Compared to a standard incubator, an onstage incubator allows optical access to the culture system on a microscope to enable real-time imaging. We interrogated two randomly selected donor neutrophils to study neutrophil activation and kinetics between these incubator conditions. Culture in the onstage incubator led to dramatically accelerated kinetics (Supplementary Fig. 8b) compared to the standard incubator. The incubation phase (see the definition in Fig. 3a) was dramatically shorter in the onstage incubator for both donors (~ 5 h) and reached the stationary phase (~ 25 h) much quicker than their standard incubator counterparts (~ 50 h) (Supplementary Fig. 8b). Therefore, we chose to use a standard incubator in this work. For experimental workflows, the culture plate was stored in a standard incubator and was only taken out for imaging in the onstage incubator.

Finally, we investigated the possible influence of cell seeding density on neutrophil kinetics. In a standard 384-well plate, 60,000 (or 60 K) cells per well achieves a near-confluent monolayer of neutrophils (Supplementary Fig. 9a). Neutrophil seeding densities across a broad range (10 K, 30 K, 60 K, and 200 K per well) were trialed. When seeded at 10 K or 30 K cells per well, neutrophils did not show any difference in normalized activation (Supplementary Fig. 9b). The highest seeding density (200 K cells per well) showed higher total activation (~ twofold higher) compared to the 60 K cells per well condition but lower activation (~ 60%) after normalization to per 10 K cells (Supplementary Fig. 9c). Overall, the near and less confluent seeding densities showed no significant influence on non-specific activation of neutrophils and the seeding density of 60 K cells per well allowed more even and consistent distribution of the cells in a well from cell seeding and thus enabled easier/more reliable data collection and analysis.

### Statistics of whole blood handling time

**Figure 2:**
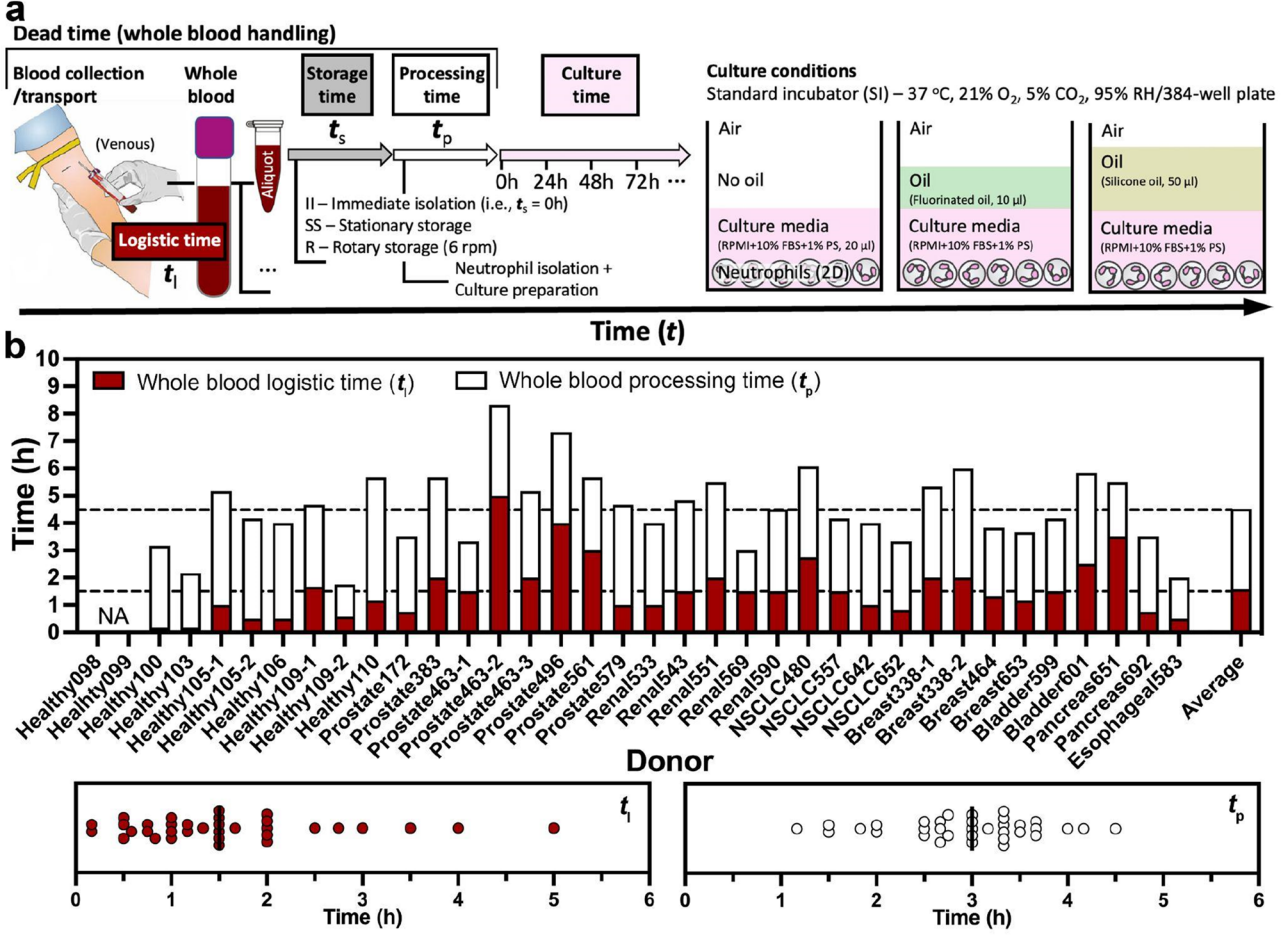
Whole blood logistics and ex vivo neutrophil interrogation in 2D cell culture. (**a**) A schematic visualizes the workflow of whole blood handling and culture conditions in this study. In a typical workflow, whole blood was collected from a donor at a designated location (e.g., hospital, clinic) and then transported to the lab for analysis. Before interrogation, the whole blood sample may be stored with different amounts of time, varying temperatures (RT versus 37 °C), and storage methods (stationary versus rotary), and then processed (i.e., neutrophil isolation and culture preparation). The 2D cell culture was performed on a 384-well plate in a standard CO_2_ incubator (“Methods” section) with and without oil overlay^29^ [for minimized media loss via evaporation/culture environmental variability, fluorinated oil (Fluorinert FC-40, highlighted in mint color)—high gas permeability, silicone oil (highlighted in tan color)—adjustable gas permeability]. (**b**) Distribution of the dead time from blood collection to the start of neutrophil interrogation. It includes whole blood logistic time (*t*_l_, the time interval between blood collection and delivery), storage time (*t*_s_), and processing time (*t*_p_, the total time of neutrophil isolation and culture plate preparation). The average *t*_l_ and *t*_p_ in this work (performed by a single operator) are about 1.5 h and 3 h, respectively.

To study human neutrophils ex vivo, the typical workflow includes blood logistics (i.e., collection and transport), storage, processing, and interrogation (e.g., cell culture and assessment) (Fig. 2a). Each additional step before interrogation in the workflow introduces possible bottlenecks and delays that can contribute to the overall quality of the isolated cells used in the terminal assay. We evaluated the components of whole blood handling to identify key parameters that influence baseline neutrophil apoptosis and NETosis, defined as “non-specific activation” after transfer to standard 2D cell culture. In the 2D culture, neutrophils were cultured in a standard incubator with and without oil overlays as described above (Fig. 2a). Blood was obtained from multiple sources including draws completed in our laboratory and through a collaborator’s lab at the UW hospital (under an IRB approved protocol, see “Methods” section). Across 36 unique blood donors, our results highlight a highly variable whole blood logistic time (*t*_l_) and whole blood processing time (*t*_p_) from case to case (Fig. 2b). *t*_l_ ranged from 0.5 to 5 h depending on the efficiency of donor ID de-identification, the distance/communication between the blood collection locus and the lab performing the subsequent experiments (Fig. 2b). Similarly, *t*_p_ ranged from 1 to 4.5 h depending on how many whole blood samples processed in an experiment and how many conditions in the following interrogation (Fig. 2b). On average, it took 4–5 h from blood collection to the start of neutrophil interrogation if no whole blood storage time (*t*_s_) was added to the “dead time” (i.e., *t*_l_ + *t*_s_ + *t*_p_) in this work performed by a single operator.

### Short-time (< 8 h) whole blood storage

Clinical studies often require processing of blood samples but can be delayed due to the logistics of transport to third-party entities, so there is a need to better understand if whole blood storage time and conditions can affect the baseline characteristics of isolated cells. Recent work identified that whole blood storage time of samples drawn from pigs led to differences in ROS production, the level of NET formation, altered neutrophil survival kinetics, and modified antimicrobial activity^31^. Here, we look into how whole blood storage time, temperature, and method (i.e., stationary storage versus rotary storage) affect apoptosis and NETosis from samples collected from healthy human donors and patients with cancer. The analysis was broken into two distinct time periods, < 8 h (i.e., short-time storage) and > 24 h (i.e., long-time storage). Early experiments indicated that the 8 h mark constituted an inflection point at which neutrophil behavior could begin to change and coincided with a traditional 8 h work shift.

**Figure 3:**
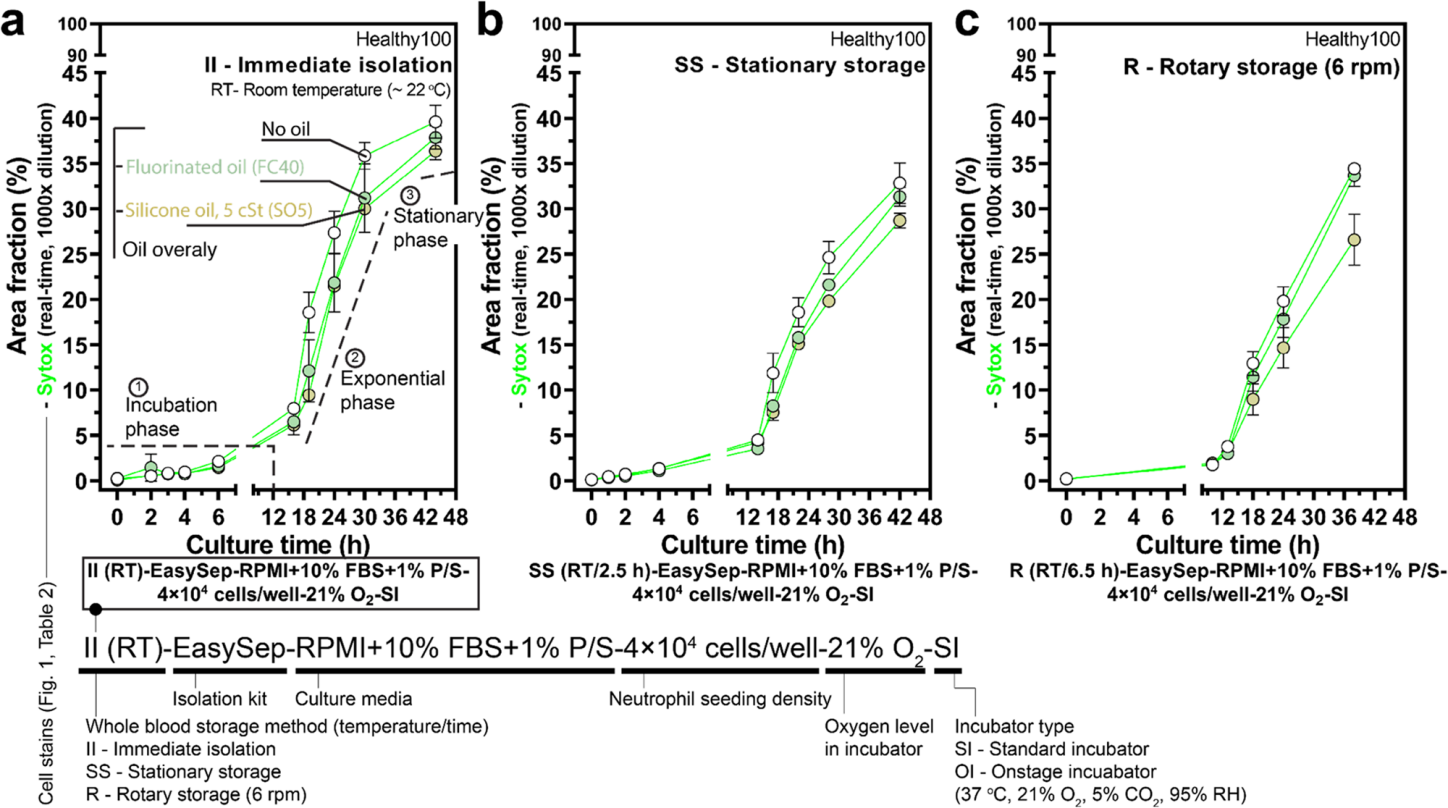
Short-time whole blood storage (*t*_s_ < 8 h) at RT in either stationary or rotary storage has little effect on non-specific activation and kinetics. (**a**) Influence of short-time whole blood storage (*t*_s_ < 8 h) at RT on non-specific activation and neutrophil kinetics in 2D culture. Results show area fraction (Supplementary Fig. 3) from the Sytox (real-time, 1000× dilution) channel (Supplementary Fig. 1a,b). The typical kinetic profile consists of incubation phase (activation < 5%), exponential phase (fast activation), and stationary phase (saturated activation). Measurements of area fraction (%) for both (**b**) stationary storage and (**c**) rotary storage along with three culture conditions [standard, i.e., no oil (white dots), fluorinated oil overlay (mint), silicone oil overlay (tan)] are not appreciably different in neutrophil activation but show less overall activation with silicone oil overlay (Supplementary Fig. 7). The plots reflect the mean ± s.d.

Considering the varying dead time of whole blood handling, it is important to understand first how short-time whole blood storage (with *t*_s_ < 8 h) may influence non-specific activation and neutrophil kinetics in standard 2D monoculture ex vivo (Fig. 3). From a whole blood sample with *t*_l_ < 1 h, we partitioned the blood into three groups: immediate isolation (i.e., *t*_s_ = 0 h) (Fig. 3a), stationary storage (at RT for 2.5 h) (Fig. 3b), and rotary storage (on a tube rotator at RT for 6.5 h) (Fig. 3c). Comparison between the stationary storage and rotary storage is limited by the 4 h time difference in storage time. This difference is attributed to practical limitations of a single operator carrying out all the tasks and material limitations (e.g., magnetic isolation kits) available to the operator. We further analyzed the kinetic profile of the isolated neutrophils after culture in the unique culture conditions. In standard 2D monoculture, the isolated neutrophils showed three distinct phases in kinetics, which include (i) incubation phase (low activation, 0–12 h), (ii) exponential phase (fast activation, 12–42 h), and (iii) stationary phase (saturated activation, > 42 h) (Fig. 3a). Our results indicate short whole blood storage times (< 8 h) did not increase non-specific activation or significantly alter kinetics in 2D culture (Fig. 3).

### Long-time (> 24 h) whole blood storage

**Figure 4:**
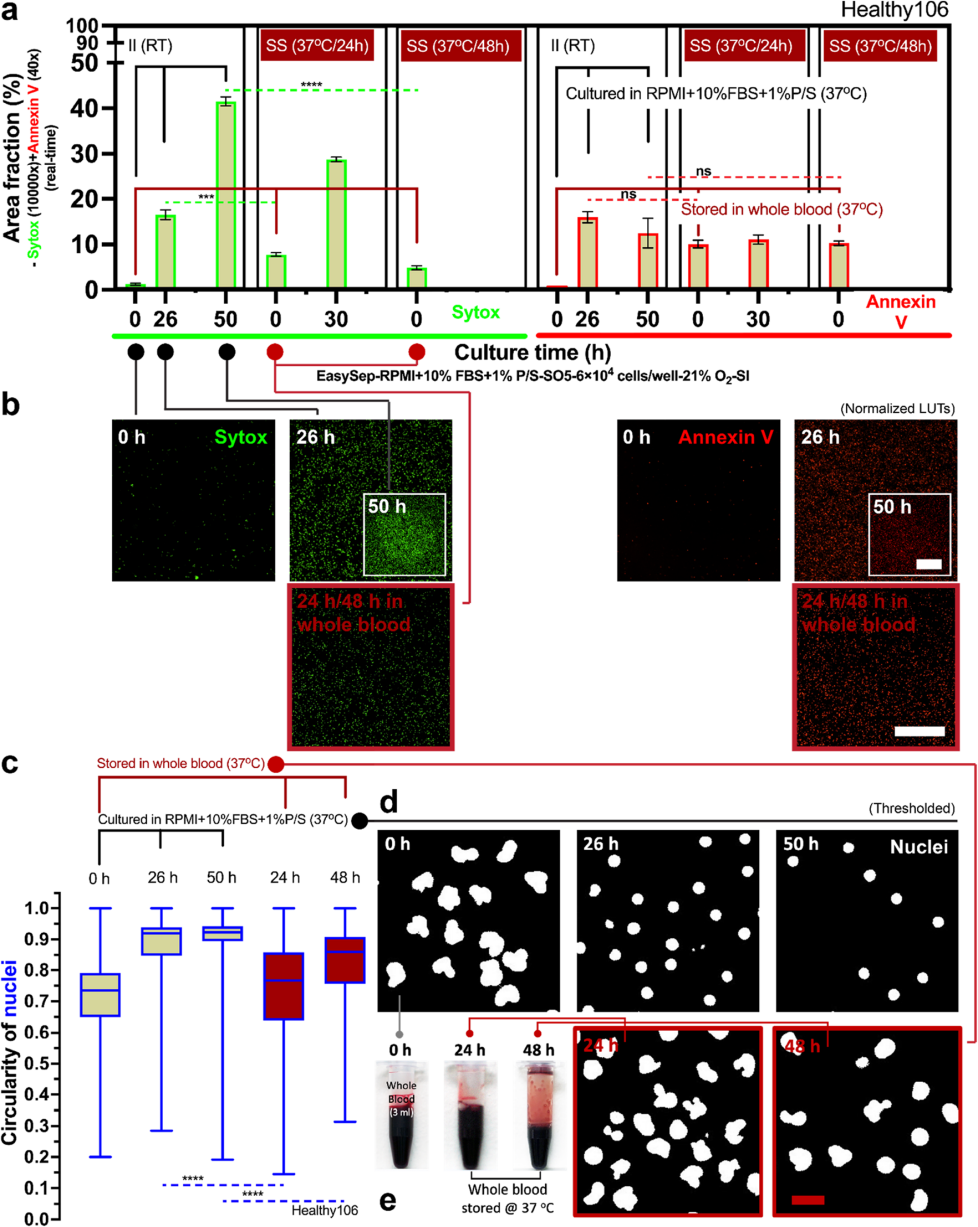

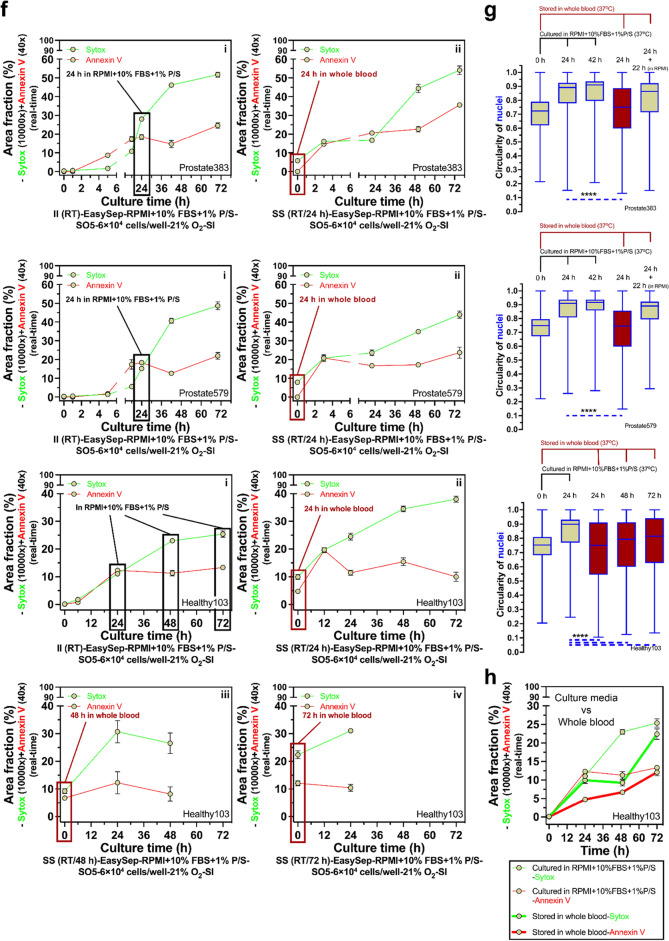
Long-time whole blood storage (*t*_s_ > 24 h) at 37 °C leads to non-specific activation and accelerated kinetic profiles. (**a**) Results show area fraction from the Sytox (real-time, 10,000 × dilution) and Annexin V (real-time, 40 × dilution) channels (Supplementary Fig. 3). The solid black line indicates neutrophil kinetics in 2D culture (RPMI + 10% FBS + 1%P/S, 37 °C). The solid dark red line indicates neutrophil kinetics stored in whole blood (37 °C). The dashed lines show the statistical significance pairs (neon green—Sytox; bright red—Annexin V) of the conditions with the same/similar time after the initiation of culture. (**b**) The representative fluorescent microscopic images show the activated cells at different conditions and time points in (**a**). Scale bar, 500 μm. (**c**) Results show circularity of nuclei (Supplementary Fig. 2). (**d**) The representative fluorescent microscopic images show the shape of nuclei at different conditions and time points in (**c**). Scale bar, 10 μm. (**e**) The color of whole blood in storage, which indicates retention of the physiological oxygen level of venous blood (~ 5% O_2_) (Supplementary Fig. 10). (**f**) Comparison of neutrophil kinetics in 2D culture on the cells isolated from whole blood at different storage times [i.e., *t*_s_ = 0 h for immediate isolation (II) versus *t*_s_ = 24 h, 48 h, and 72 h in whole blood). (**g**) The results of circularity of nuclei in (**f**). (**h**) Side-by-side comparison of neutrophil kinetics between 2D culture (thin lines) and whole blood storage (thick lines). The 2D cultures were all performed with silicone oil (5 cSt, abbreviation “SO5”, coded in tan color) overlay. These data are representative quantification of one of the n = 3 donors with at least n = 3 technical replicates for each donor. The data for (**a,f**) reflects the mean ± s.d. The data for (**c,g**) are visualized by a box and whisker plot. *P ≤ 0.05, **P ≤ 0.01, ***P ≤ 0.001, and ****P ≤ 0.0001. “ns” represents “not significant” (One-way ANOVA + Tukey’s post-hoc test).

Conversely, long-time whole blood storage times (*t*_s_ > 24 h) may influence non-specific activation of neutrophils (Fig. 4). Neutrophils stored at 37 °C, in a standard 2D monoculture environment, for greater than 24 h and up to 48 h showed increased levels of Sytox staining (Fig. 4a,b), which accounts for NETosis and secondary (sec.) necrosis (Fig. 1). In contrast, neutrophils stored in whole blood at 37 °C maintain relatively low levels of Sytox staining immediately after isolation, indicating low levels of non-specific activation (Fig. 4a,b). In all conditions, Annexin V staining, which measures apoptosis, sec. necrosis, and suicidal NETosis (Fig. 1), was not appreciably different. As cells undergo apoptosis and nuclear condensation occurs, the circularity of the multilobed neutrophil nucleus increases towards 1.00 (which indicates a round nucleus) (Supplementary Fig. 2). The automated nuclear circularity analysis pipeline (“Methods” section) developed here quantified the differences in circularity between the different storage conditions at the specified time points. Neutrophils that were immediately isolated and kept in culture at RT had increased circularity (i.e., more apoptosis) over the subsequent 48 h of culture (Fig. 4c,d). Neutrophils stored in whole blood at 37 °C and subsequently isolated at the 24 h and 48 h time points had significantly less circularity (i.e., more healthy neutrophils with multilobed nucleus) when compared to their RT counterparts (Fig. 4c,d).

Next, we investigated the kinetic profiles of neutrophils in the different storage conditions and length of storage. For these studies, each whole blood sample, from three random donors (Prostate383, Prostate579, Healthy103), was partitioned into four groups—immediate isolation, and whole blood storage for 24 h, 48 h, and 72 h. Neutrophils in the immediate isolation condition were placed in 2D culture and incubated in RPMI + 10% FBS + 1% Penicillin/Streptomycin (P/S) (Fig. 4f Prostate383-i, Prostate579-i, Healthy103-i). These neutrophils were monitored for the subsequent 72 h to assess their kinetic profile. In two of the donors (Prostate383, Prostate579), the neutrophils stored in whole blood for 24 h were subsequently isolated and placed into 2D culture as described above for 72 h (Fig. 4f Prostate383-ii, Prostate579-ii) to assess their kinetic profiles. For the third donor (Healthy103), neutrophils stored in whole blood for 24 h, 48 h, and 72 h were subsequently isolated and placed into 2D culture as described above (Fig. 4f Healthy103-ii, Healthy103-iii, Healthy103-iv). For each donor, the circularity of nuclei of the neutrophils was measured to assess apoptosis (Fig. 4g) across the different time points and storage conditions. Neutrophils stored in whole blood for 24 h prior to isolation showed accelerated kinetics (Fig. 4f Prostate383-ii, Prostate579-ii, Healthy103-ii) compared to the immediate isolation control (Fig. 4f Prostate383-i, Prostate579-i, Healthy103-i) as their 0 h time point showed significantly higher levels of non-specific activation (~ 10% vs 0%). Furthermore, neutrophils from the third donor (Healthy103) that were placed into culture at 48 h and 72 h post-whole blood storage showed even greater levels of kinetic acceleration, since they reached the stationary phase of their activation profile early on in 2D culture (Fig. 4f Healthy103-iii, Healthy103-iv). However, neutrophils stored in whole blood for 24 h had lower levels of apoptosis, as measured by nuclear circularity, compared to the 24 h time point for the immediate isolation condition for all three donors (Fig. 4g). For the third donor, the neutrophils stored in whole blood for 24 h, 48 h, and 72 h all show less apoptosis compared to the 24 h time point of the immediate isolation condition (Fig. 4g). For the third donor, we compared the level of activation of the neutrophils in each storage condition with respect to total time after isolation instead of time in 2D culture. Neutrophils in either 2D culture with RPMI + 10% FBS + 1% P/S or stored in whole blood showed similar levels of non-specific activation and apoptosis up till 24 h after isolation, but there were significantly lower levels of non-specific activation and apoptosis in the whole blood storage condition between 24 to 48 h time points (Fig. 4h). By 72 h, both storage conditions showed high levels of neutrophil non-specific activation and similar levels of apoptosis (Fig. 4h).

### Whole blood storage temperature

**Figure 5:**
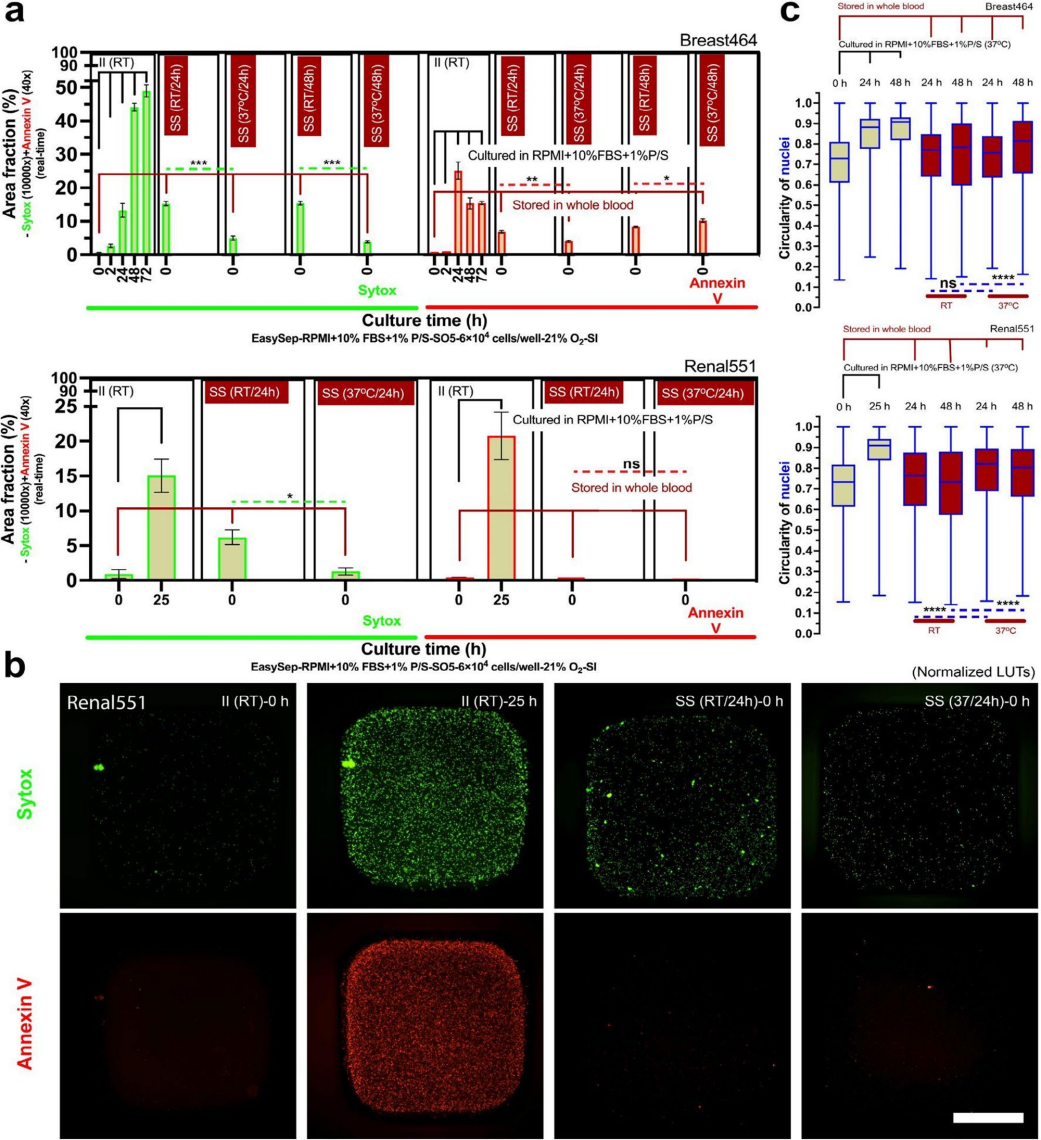
Storage temperature (RT versus 37 °C) in long-time whole blood storage on non-specific activation and kinetics. (**a**) Results show area fraction from the Sytox (real-time, 10,000× dilution) and Annexin V (real-time, 40× dilution) channels. Non-specific activation of neutrophils increased over 72 h in 2D culture. Neutrophils stored in whole blood at 37 °C showed less activation than the RT storage condition at both 24 h and 48 h. Apoptosis however does not show a clear trend with respect to storage temperature. (**b**) Representative images of Sytox and Annexin V staining for both whole blood storage temperatures seen in (**a**). Scale bar, 1 mm. (**c**) Nuclei circularity analysis shows more apoptosis in the 37 °C whole blood storage condition compared to the corresponding RT condition at both 24 h and 48 h. These data are representative quantification of one of the n = 3 donors with at least n = 3 technical replicates for each donor. The data for (**a**) reflects the mean ± s.d. The data for (**c**) are visualized by a box and whisker plot. *P ≤ 0.05, **P ≤ 0.01, ***P ≤ 0.001, and ****P ≤ 0.0001. “ns” represents “not significant” (One-way ANOVA + Tukey’s post-hoc test).

Finally, we investigated the influence of whole blood storage temperature on non-specific activation and neutrophil kinetics. Across two donors, whole blood was partitioned into immediate isolation, and either storage at RT or 37 °C. Neutrophil activation and apoptosis were measured at 0 h, 2 h, 24 h, 48 h, and 72 h for the immediate isolation condition and at 24 h and 48 h for the RT and 37 °C storage conditions. For the second donor, technical limitations prevented us from capturing the 48 h time point in the two storage temperature conditions. In the immediate isolation condition, neutrophil activation increased over the 72 h of culture with a parallel increase in Annexin V staining, which measures early apoptosis, sec. necrosis, and suicidal NETosis. The four storage conditions have higher levels of Sytox and Annexin V staining at 0 h time point compared to the immediate isolation control, however, the neutrophils stored at 37 °C for 24 h and 48 h both had lower levels of Sytox staining compared to their matched RT storage conditions (Fig. 5a,b). Annexin staining between the immediate isolation condition and both storage conditions was not consistent between the two donors. For both donors, nuclei circularity analysis showed that there was significantly more apoptosis in the 37 °C storage condition compared to the RT storage condition (Fig. 5c).

## Discussion

Neutrophils are ubiquitous in circulation and their unique roles in cellular immunity continue to be elucidated. In recent years, their role in a variety of disease states has become more apparent which coincides with increased usage of neutrophils ex vivo for interrogation in a breadth of functional assays and molecular characterizations. Delays in neutrophil interrogation from time of whole blood collection to final assay processing are abundant due to inherent logistical hurdles, and potentially elongating factors associated with transport to end-user facilities. Heterogeneity in neutrophil biology can be attributed to a host of factors including the underlying disease state, intrinsic patient-to-patient differences, and as shown here—modifiable external factors after whole blood collection and prior to final interrogation in an assay of interest. Comprehensive characterization of neutrophils ex vivo has also been limited by poor ex vivo stability leading to non-specific activation. High variability in the baseline non-specific activation of neutrophils introduces variability that can make technical replicates and experimental replicates challenging to interpret or compare. Here we sought to elucidate how some of these factors—specifically, time from collection prior to isolation, storage orientation, time, and temperature influence baseline characteristics of isolated neutrophils in standard 2D cell culture. The wet-lab work for this study was completed by a single operator to reduce potential person-to-person variability in whole blood handling and cell culture.

Using an optimized trio of dyes (Hoechst, Sytox, Annexin V), we characterized how the above modifiable factors contribute to the non-specific activation state of isolated neutrophils by measuring apoptosis and NETosis through an automated image analysis pipeline across subsets of 36 unique blood donors. We identified that the EasySep negative selection method without RBC lysis yielded abundant neutrophils with minimal non-specific activation and normal kinetic profiles. Based on our experience with microscale cultures in microfluidic devices we were cognizant of the influence of evaporation, culture environment, and seeding density on cell behavior^25–27,29^. We observed that minimizing evaporation with a layer of silicone oil on top of normal cell culture media decreased non-specific activation of neutrophils from some donors. Furthermore, we observed that 2D culture in the standard CO_2_ incubator showed more consistent and minimal neutrophil activation compared to an on-stage microscope incubator. Traditional incubators are better sealed and are less prone to shifts in O_2_ levels, CO_2_ levels, temperature, and RH in contrast to on-stage microscope incubators.

Through processing samples from healthy donors and patients with cancer across two collection sites, we observed that the average dead time (i.e., whole blood logistic time + storage time + processing time) from a single operator was about 5 h. Despite both collection sites being located within the same building, the high variability in dead time highlighted the need to better understand how even more disparate conditions (e.g., cross campus or cross institute delivery) could affect neutrophil behavior after storage in whole blood. Maintaining the whole blood in a rotary configuration was hypothesized to impose shear stress on the cells to better mimic the in vivo physiology of vasculature and hopefully improve neutrophil stability; however, whole blood storage (stationary or rotary) within 8 h (at RT) did not cause appreciable differences in non-specific activation and neutrophil kinetics in standard 2D monoculture.

For storage beyond 24 h, neutrophils stored in whole blood (at 37 °C) showed significantly higher stability (i.e., less non-specific activation) compared to the 2D culture controls. It is worth noting that we observed that long-time whole blood storage leads to decreased neutrophil yield from negative selection-based isolation methods. Additionally, neutrophils stored at 37 °C had less NETosis but had increased apoptosis compared to RT. While neutrophils can maintain low non-specific activation in whole blood up to 48 h, a storage time > 48 h in whole blood leads to non-specific activation at a level comparable to the cells in standard 2D monoculture for the same length of time. Neutrophils stored in whole blood for between 24 and 48 h also exhibited accelerated kinetics when placed in 2D culture compared to the immediate isolation control. Assessing neutrophil storage across multiple days highlights the importance of evaluating both the activation status and kinetics when trying to assess the baseline status of neutrophils. Despite showing minimal activation across all the long-time storage conditions (up to 72 h), which would suggest that any of these cells would be amenable to use in an ex vivo assay, the acceleration of the kinetic profiles of the neutrophils stored in whole blood > 24 h would introduce significant heterogeneity into a study and make potential functional readouts less comparable between experimental and biological replicates.

## Study limitations

A limitation of this study is that only apoptosis and NETosis are studied while other functional readouts like ROS production or antimicrobial activity and molecular readouts are not analyzed. These differences would also provide insight into how whole blood logistics and short storage time conditions could affect these readouts; however, the observed fundamental differences in the phenotypic markers of baseline non-specific activation and kinetics over time we identified highlight the need to prioritize and account for these factors before more expensive and detailed characterization is completed. Furthermore, we did not further stratify the analysis by donor age or clinical status due to limitations in what information we can collect per our IRB protocol. However, future studies would benefit from an in depth analysis of these variables since immune dysfunction, including innate immunity, is present at baseline for cancer patients and especially after treatment with common therapeutic agents^32,33^.

## Conclusions

All together our data illustrates the potential effects that externally modifiable factors can have on neutrophil non-specific activation and kinetics. Whole blood logistics are highly variable, and this variability will be unique to each institution and affiliated third-party users who perform the end readouts. Our data suggest that minimizing the time from collection to assay to less than 8 h can help to improve reproducibility of readouts, since we observed no significant differences in non-specific activation or kinetics of the neutrophils at these timepoints. Additionally, whole blood storage at 37 °C for up to 48 h can maintain the stability of neutrophils with minimal activation but with measurable increases in neutrophil kinetics. While several important parameters focusing on whole blood logistics are investigated here, additional variables related to the physiological factors in the in vivo environment are not discussed, including co-culture with common neighboring in vivo cell types (e.g., endothelial cells), oxygen tension (e.g., normoxia and hypoxia), and autologous signaling molecules (e.g., cytokines, hormones, growth factors). These factors will likely also influence the overall quality of isolated neutrophils from whole blood. In addition to results reported above, inclusion of these parameters will lead to a more complete picture of the parameter space that is essential for maintaining in vivo-like neutrophil characteristics ex vivo to generate robust and reliable readouts of neutrophil behavior. We believe these efforts reported here provide a template for how other variables can be assayed to achieve a high-throughput, quality control method for sample analysis and measurement of phenotypic markers of non-specific activation of neutrophils prior to interrogation in an ex vivo assay.

### Supplementary Information

Supplementary Figures.

## Data Availability

The data that support the findings of this study are available from the corresponding authors upon reasonable request.

## Code availability

All code used to analyze data and produce figures has been uploaded to Github—https://github.com/jcai0791/Neutrophil-Project.

## References

[1] Grecian R, Whyte MKB, Walmsley SR (2018). The role of neutrophils in cancer. Br. Med. Bull..

[2] Kobayashi SD, Malachowa N, DeLeo FR (2018). Neutrophils and bacterial immune evasion. J. Innate Immunity.

[3] Hidalgo A, Chilvers ER, Summers C, Koenderman L (2019). The neutrophil life cycle. Trends Immunol..

[4] Cartwright GE, Athens JW, Wintrobe MM (1964). The kinetics of granulopoiesis in normal man. Blood.

[5] Pillay J (2010). In vivo labeling with 2H2O reveals a human neutrophil lifespan of 5.4 days. Blood.

[6] Tofts PS, Chevassut T, Cutajar M, Dowell NG, Peters AM (2011). Doubts concerning the recently reported human neutrophil lifespan of 5.4 days. Blood.

[7] Lahoz-Beneytez J (2016). Human neutrophil kinetics: Modeling of stable isotope labeling data supports short blood neutrophil half-lives. Blood.

[8] Brinkmann V (2004). Neutrophil extracellular traps kill bacteria. Science.

[9] Cahilog Z (2020). The role of neutrophil NETosis in organ injury: Novel inflammatory cell death mechanisms. Inflammation.

[10] Kenny EF (2017). Diverse stimuli engage different neutrophil extracellular trap pathways. Elife.

[11] Brodin P (2015). Variation in the human immune system is largely driven by non-heritable influences. Cell.

[12] MacGillivray DM, Kollmann TR (2014). The role of environmental factors in modulating immune responses in early life. Front. Immunol..

[13] Krabbe J (2020). Blood collection technique, anticoagulants and storing temperature have minor effects on the isolation of polymorphonuclear neutrophils. Sci. Rep..

[14] Alipour R, Fatemi A, Alsahebfosul F, Andalib A, Pourazar A (2020). Autologous plasma versus fetal calf serum as a supplement for the culture of neutrophils. BMC Res. Notes.

[15] Walmsley SR (2005). Hypoxia-induced neutrophil survival is mediated by HIF-1alpha-dependent NF-kappaB activity. J. Exp. Med..

[16] Marwick JA (2013). Oxygen levels determine the ability of glucocorticoids to influence neutrophil survival in inflammatory environments. J. Leukoc. Biol..

[17] Hind LE, Ingram PN, Beebe DJ, Huttenlocher A (2018). Interaction with an endothelial lumen increases neutrophil lifetime and motility in response to. Blood.

[18] Veda P (2011). Why are neutrophils polymorphonuclear?. Eur. J. Inflamm..

[19] Pérez-Figueroa E, Álvarez-Carrasco P, Ortega E, Maldonado-Bernal C (2021). Neutrophils: Many ways to die. Front. Immunol..

[20] Remijsen Q (2011). Dying for a cause: NETosis, mechanisms behind an antimicrobial cell death modality. Cell Death Differ..

[21] Zhao W, Fogg DK, Kaplan MJ (2015). A novel image-based quantitative method for the characterization of NETosis. J. Immunol. Methods.

[22] Jensen EC (2012). Use of fluorescent probes: Their effect on cell biology and limitations. Anat. Rec. Adv. Integr. Anat. Evol. Biol..

[23] Durand RE, Olive PL (1982). Cytotoxicity, mutagenicity and DNA damage by Hoechst 33342. J. Histochem. Cytochem..

[24] Blanter M (2022). Method matters: Effect of purification technology on neutrophil phenotype and function. Front. Immunol..

[25] Li C (2018). Exclusive liquid repellency: An open multi-liquid-phase technology for rare cell culture and single-cell processing. ACS Appl. Mater. Interfaces.

[26] Li C (2018). Double-exclusive liquid repellency (double-ELR): An enabling technology for rare phenotype analysis. Lab Chip.

[27] Li C (2020). Under oil open-channel microfluidics empowered by exclusive liquid repellency. Sci. Adv..

[28] Li C (2021). Social motility of biofilm-like microcolonies in a gliding bacterium. Nat. Commun..

[29] Li C (2022). Under-oil autonomously regulated oxygen microenvironments: A goldilocks principle-based approach for microscale cell culture. Adv. Sci..

[30] Li C, Niles DJ, Juang DS, Lang JM, Beebe DJ (2019). Automated system for small-population single-particle processing enabled by exclusive liquid repellency. SLAS Technol..

[31] Bonilla MC (2020). How long does a neutrophil live?—The effect of 24 h whole blood storage on neutrophil functions in pigs. Biomedicines.

[32] Butcher S, Chahel H, Lord JM (2000). Review article: Ageing and the neutrophil: No appetite for killing?. Immunology.

[33] Maiorino L, Daßler-Plenker J, Sun L, Egeblad M (2022). Innate immunity and cancer pathophysiology. Annu. Rev. Pathol..

