# Supplementary material for: The effect of whole blood logistics on neutrophil non-specific activation and kinetics ex vivo
Source: Sci Rep. 2024 Jan 30;14:2543. doi: 10.1038/s41598-023-50813-x (PMC10828393; doi:10.1038/s41598-023-50813-x)
Supplement: Supplementary file 1 — Supplementary Figures. [file 41598_2023_50813_MOESM1_ESM.pdf]

Supplementary Information for

The effect of whole blood logistics on neutrophil non-specific activation and kinetics *ex vivo*

Chao Li[1]*, Mehtab Farooqui[2], Ravi Chandra Yada[2,3,4], Joseph B. Cai[2], Anna Huttenlocher[5,6] & David J. Beebe[1,2,3]*

*To whom correspondence may be addressed.
email: cli479@wisc.edu (Chao Li); djbeebe@wisc.edu (David J. Beebe)

This PDF file includes:
Supplementary Figures 1-10

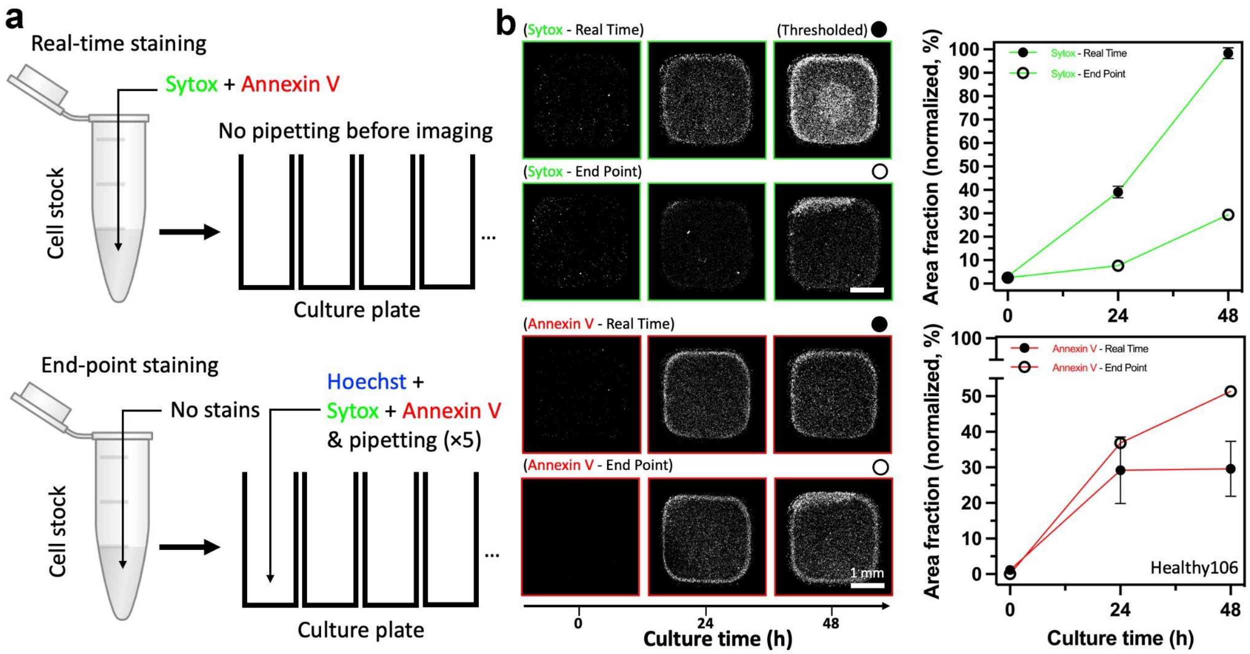
a
Real-time staining
Sytox + Annexin V
No pipetting before imaging
Cell stock
Culture plate
End-point staining
No stains
Hoechst +
Sytox + Annexin V
& pipetting (×5)
Cell stock
Culture plate
b
(Sytox - Real Time)
(Thresholded)
(Sytox - End Point)
(Annexin V - Real Time)
(Annexin V - End Point)
1 mm
0
24
48
Culture time (h)
Sytox - Real Time
Sytox - End Point
Area fraction (normalized, %)
Annexin V - Real Time
Annexin V - End Point
Healthy106
Culture time (h)

c

[II (RT)-EasySep-RPMI+10% FBS+1% P/S-6×10^4 cells/well-SO5-21% O_2-SI, neutrophil, real-time staining] **Healthy106**

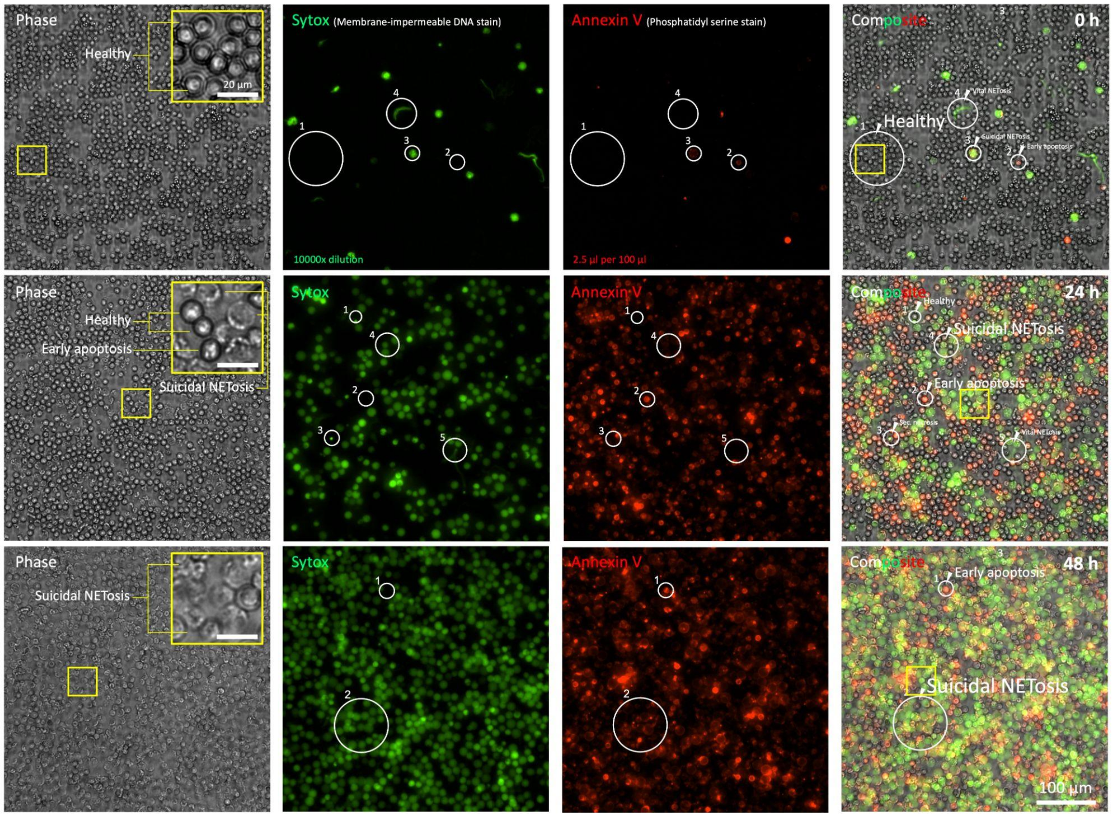

d

[II (RT)-EasySep-RPMI+10% FBS+1% P/S-6×10^4 cells/well-SO5-21% O_2-SI, neutrophil, end-point staining] **Healthy106**

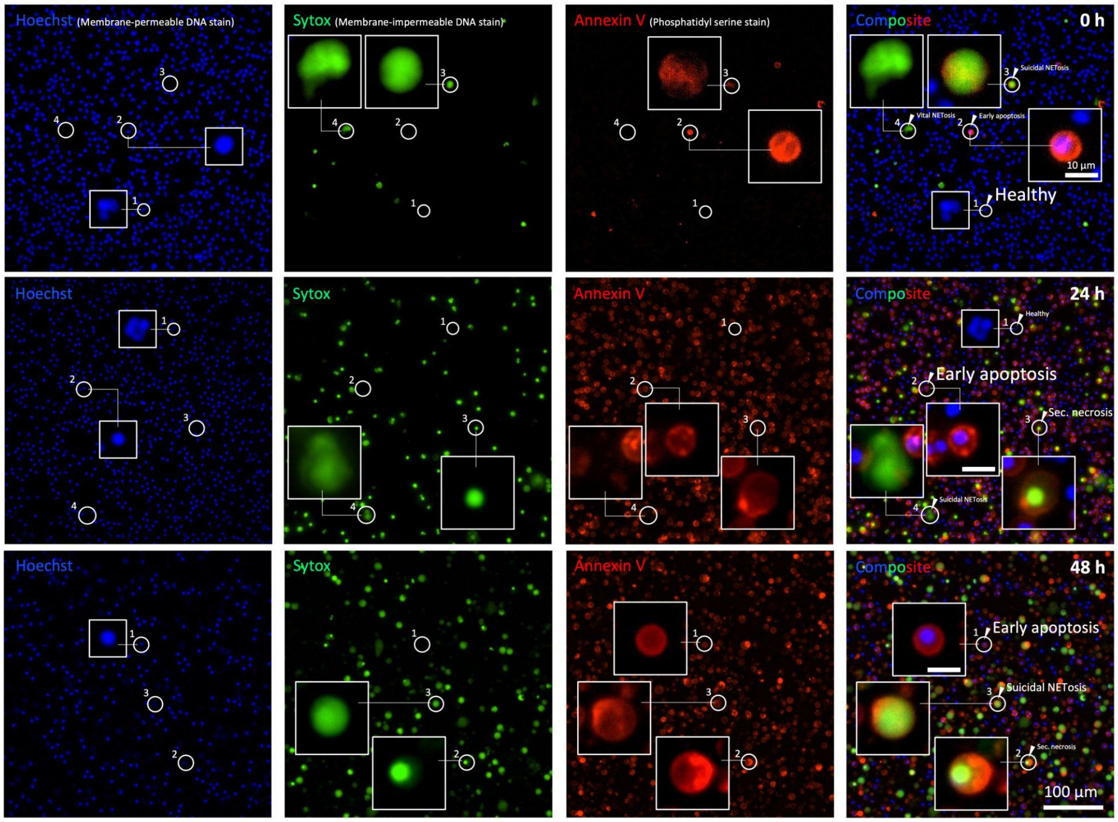

e

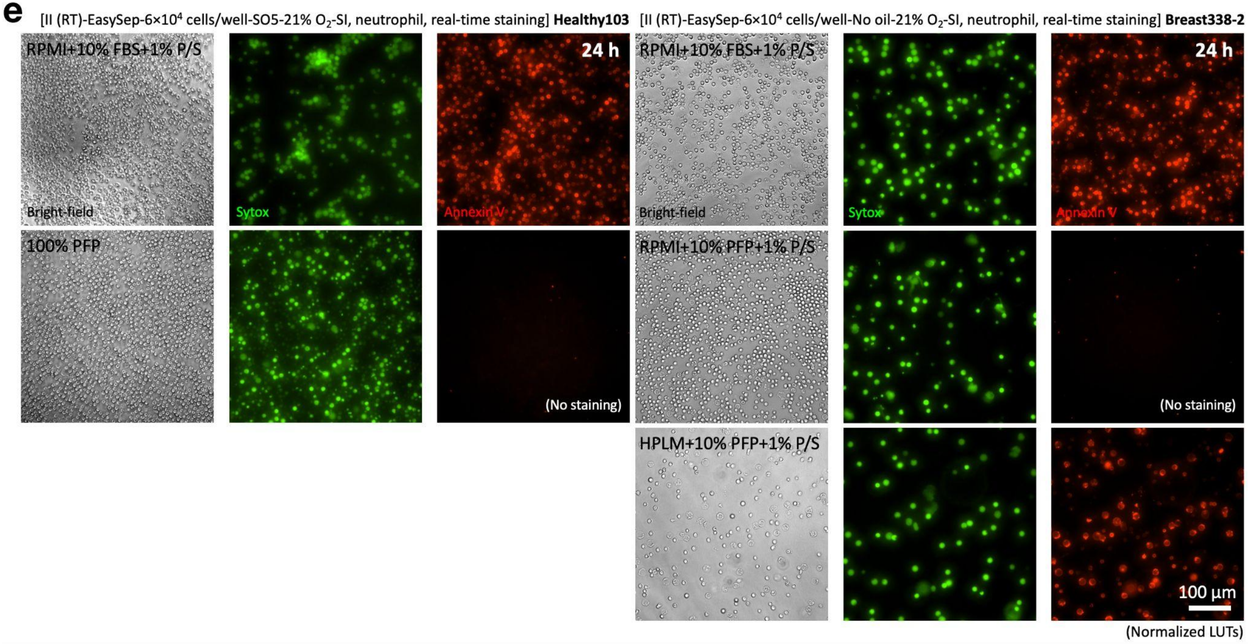

f

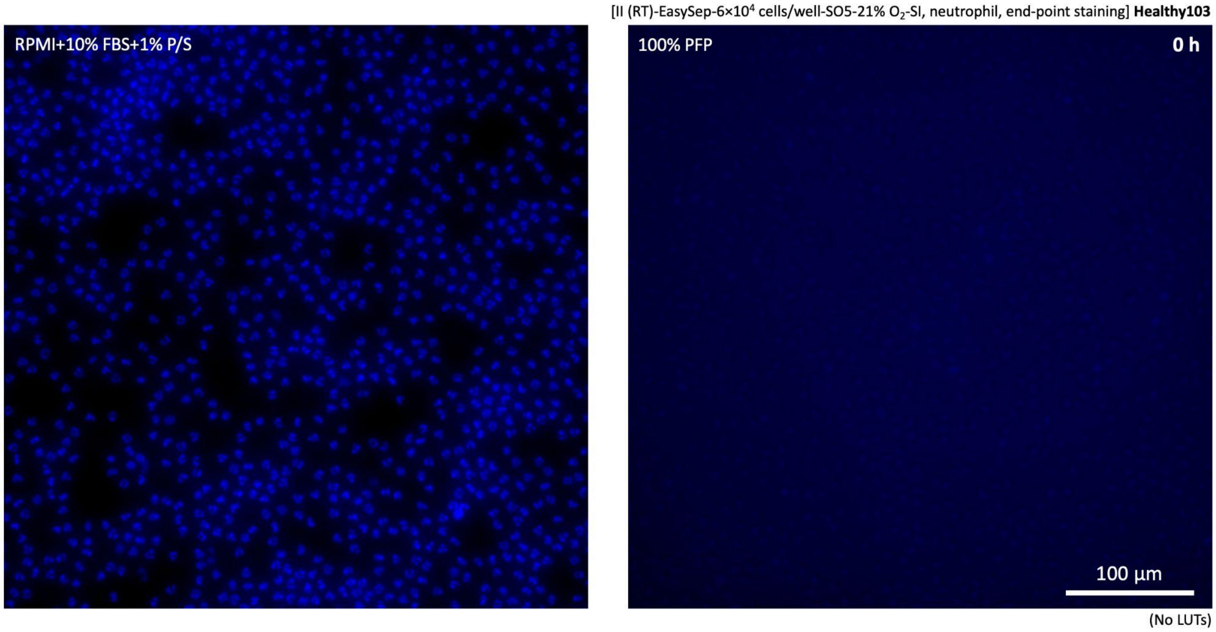

Supplementary Fig. 1 Real-time staining and end-point staining in the H-S-A method. a A schematic shows the workflow in each staining protocol. **b** Experimental results show the difference between real-time staining (positive error compared to no-dye control) and end-point staining (negative error compared to no-dye control). Real-time staining potentially inflicts irritation to the cells (especially the sensitive type) and thus may cause positive error of the fluorescence signals. By contrast, cells that undergo NETosis are fragile and break into debris from pipet mixing, and therefore, end-point staining (that requires mixing) causes negative error of the fluorescence signals. Typical microscopic images from real-time staining (**c**), and end-point staining (**d**). A summary of the function and readout of the three dyes is discussed in Table 2 in the main text. **e**, Annexin V shows little staining when used in a specific culture media (e.g., RPMI, however, no such issue found with HPLM) with human plasma additive. **f**, Hoechst shows a strong background in human plasma.

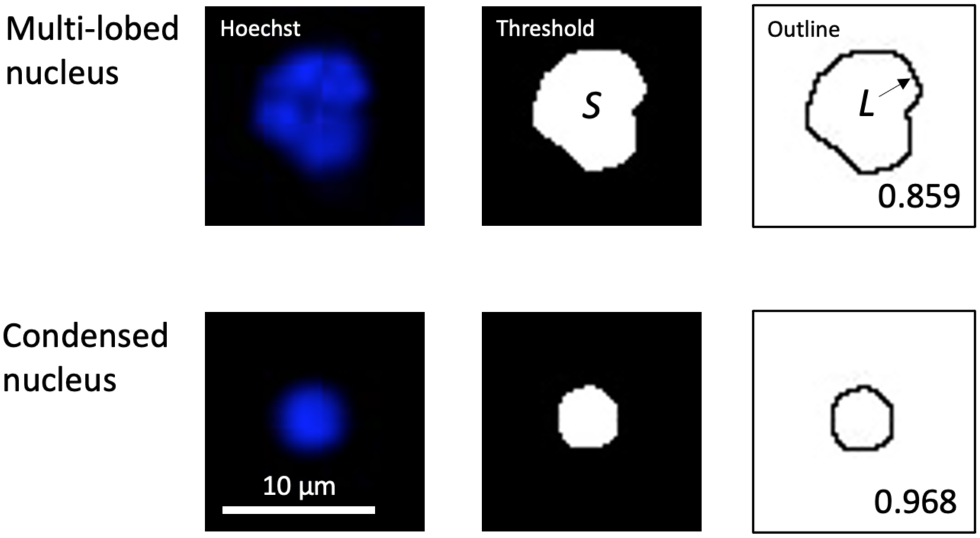

Supplementary Fig. 2 Circularity analysis for quantifying shape change of nuclei. Circularity [0.00-1.00] = $S/S_{\mathrm{circle}} = 4\pi S/L^2$, where S is the area of the particle, L is the perimeter of the particle, S_{circle} is the area of a circle with perimeter L.

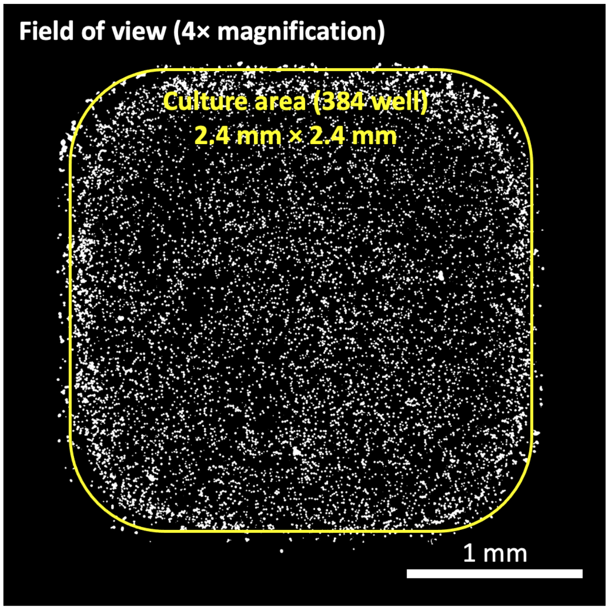

Supplementary Fig. 3 Area fraction analysis for quantifying non-specific activation (i.e. the baseline apoptosis and NETosis) of neutrophils. Non-specific activation$_{\mathrm{Sytox\ or\ Annexin\ V}}$ (%) = ($S_{\mathrm{particles}}/S_{\mathrm{culture\ area}}$) × 100% = Area fraction (Image J) × ($S_{\mathrm{field\ of\ view}}/S_{\mathrm{culture\ area}}$) × 100%, where Area fraction (Image J) = $S_{\mathrm{particles}}/S_{\mathrm{field\ of\ view}}$, S is for surface area.

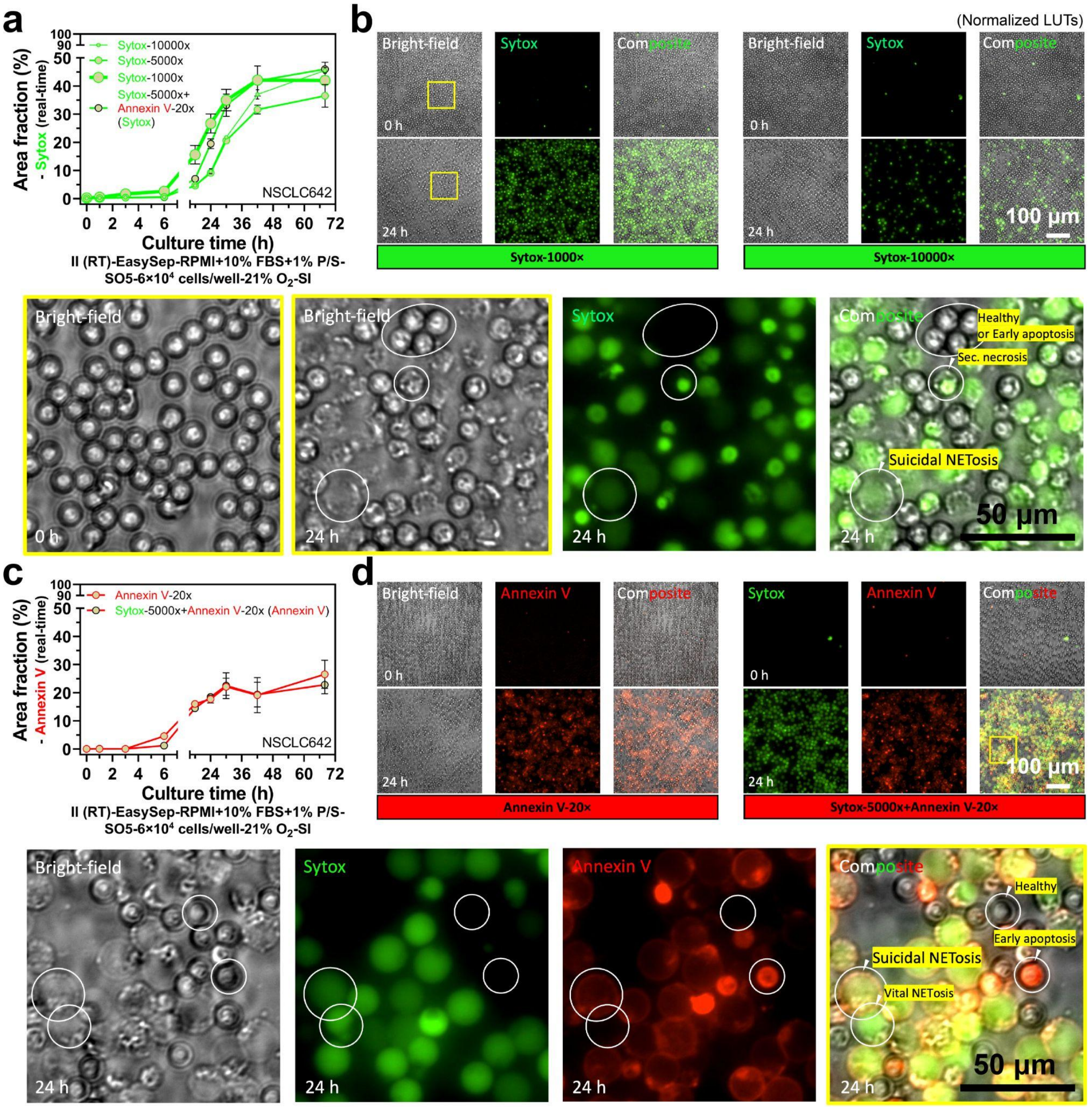

Supplementary Fig. 4 Influence of cell stains (Sytox and Annexin V, real-time staining) on non-specific activation and neutrophil kinetics in standard 2D monoculture. a Comparison of different concentrations of Sytox and the influence of addition of Annexin V to Sytox. Results are area fraction from the Sytox (real-time) channel. **b** The representative microscopic images in (**a**). **c** The influence of addition of Sytox to Annexin V. Results are area fraction from the Annexin V (real-time) channel. **d** The representative microscopic images in (**c**). The no-dye control and comparison are shown in Supplementary Fig. 5.

[II (RT)-EasySep-RPMI+10% FBS+1% P/S-6×10^4 cells/well-SO5-21% O_2-SI, neutrophil, real-time staining] **Healthy106**

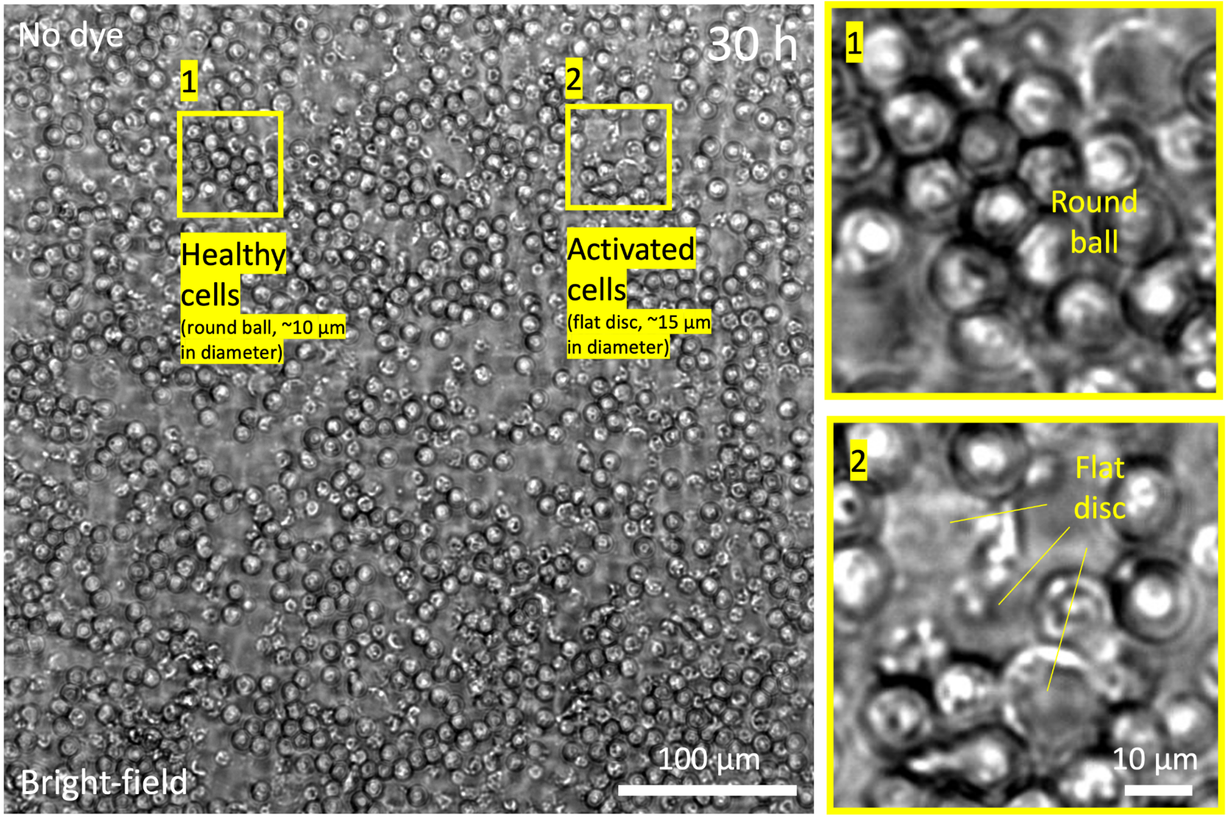

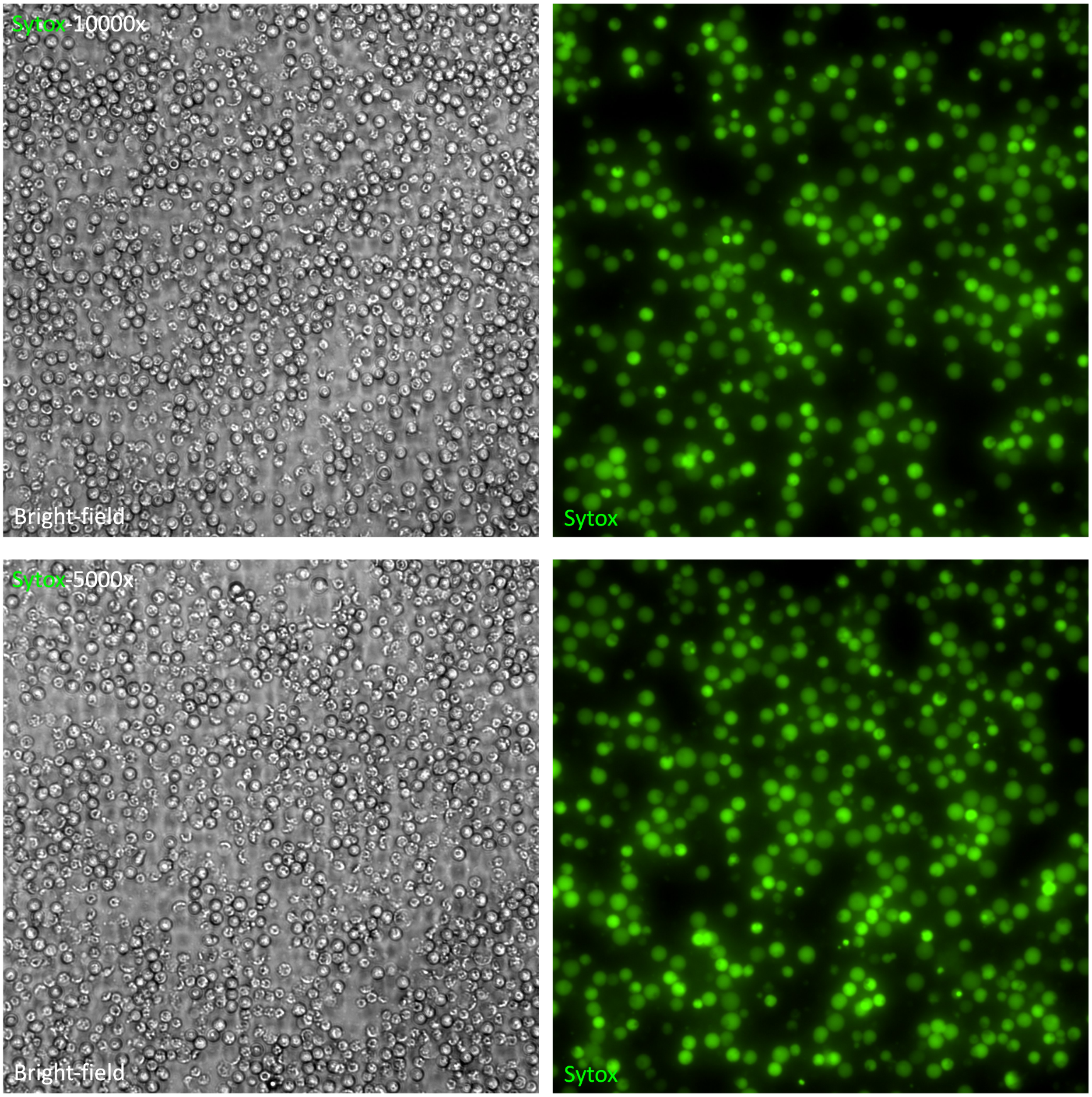
Sytox 10000x
Bright-field
Sytox
Sytox 5000x
Bright-field
Sytox

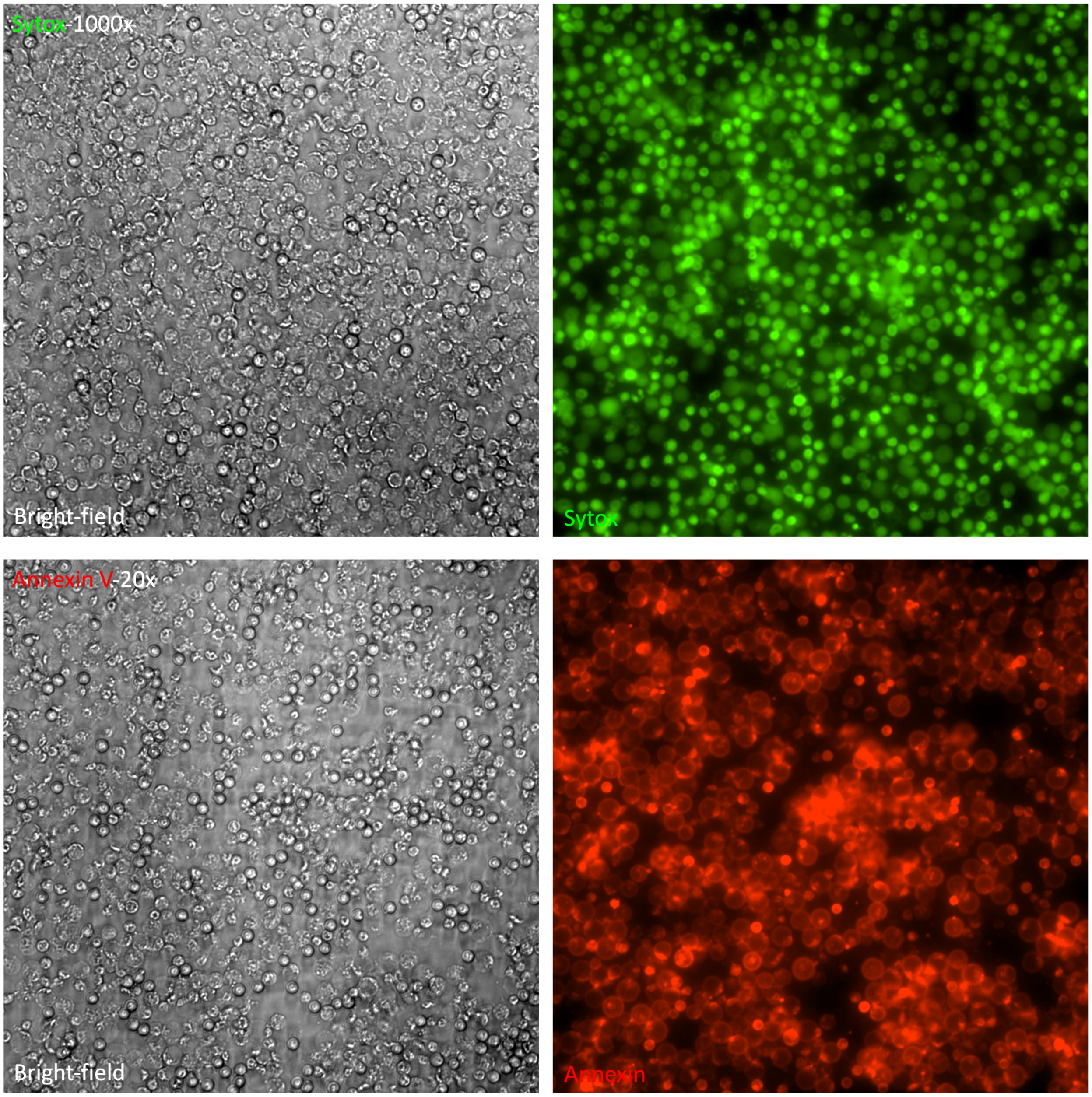
Sytox 1000x
Bright-field
Sytox
Annexin V 20x
Bright-field
Annexin

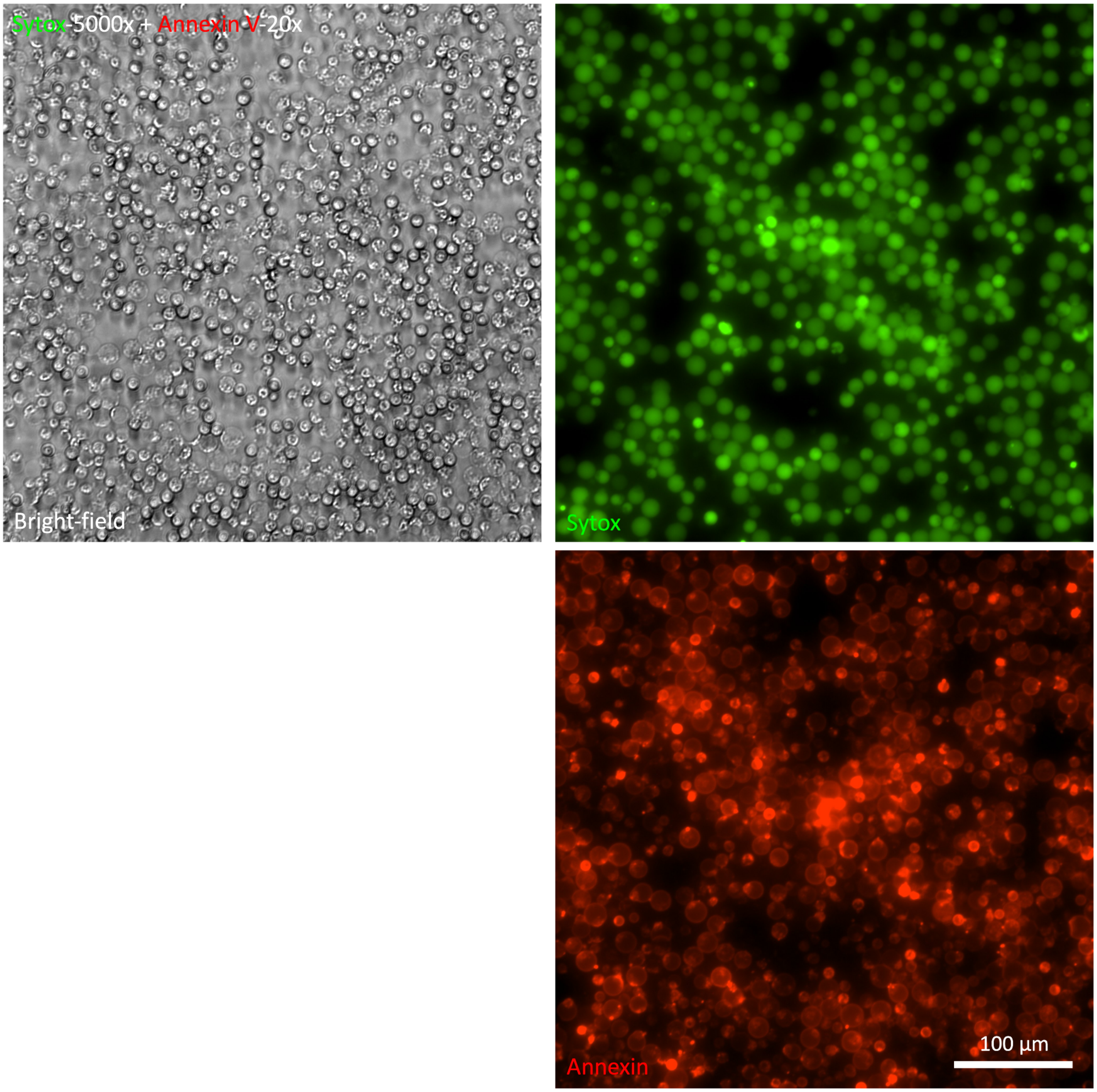

Supplementary Fig. 5 Comparison between no dye and cell stain on non-specific activation of neutrophils.

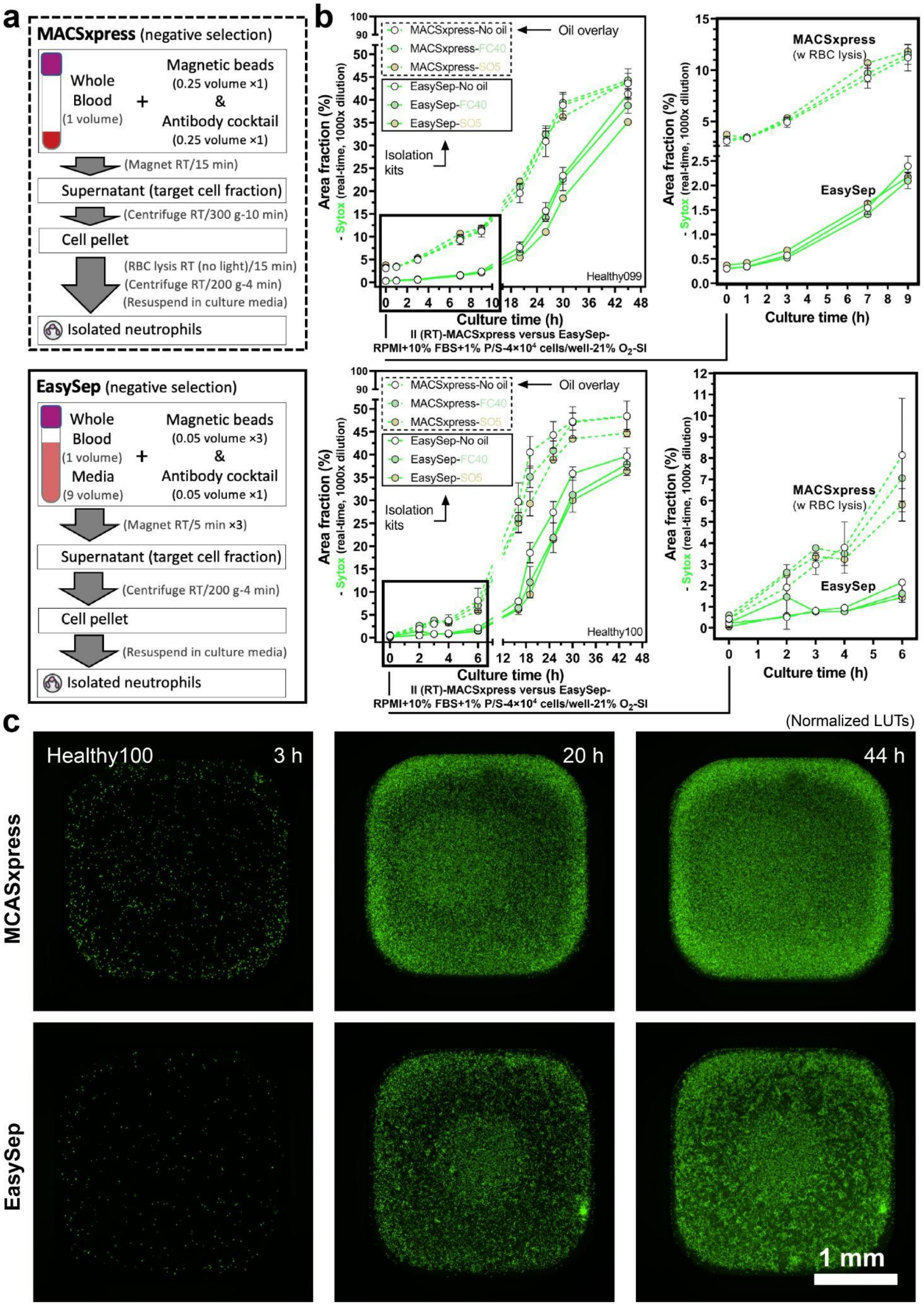

Supplementary Fig. 6 Influence of isolation method on non-specific activation and neutrophil kinetics in culture. a The workflow of two negative-selection based isolation kits - MACSxpress (top) and EasySep (bottom). **b** Comparison between the two kits on non-specific activation and neutrophil kinetics in culture. Results are area fraction from the Sytox (real-time, 1000× dilution) channel. **c** The representative microscopic images in (**b**), Healthy100.

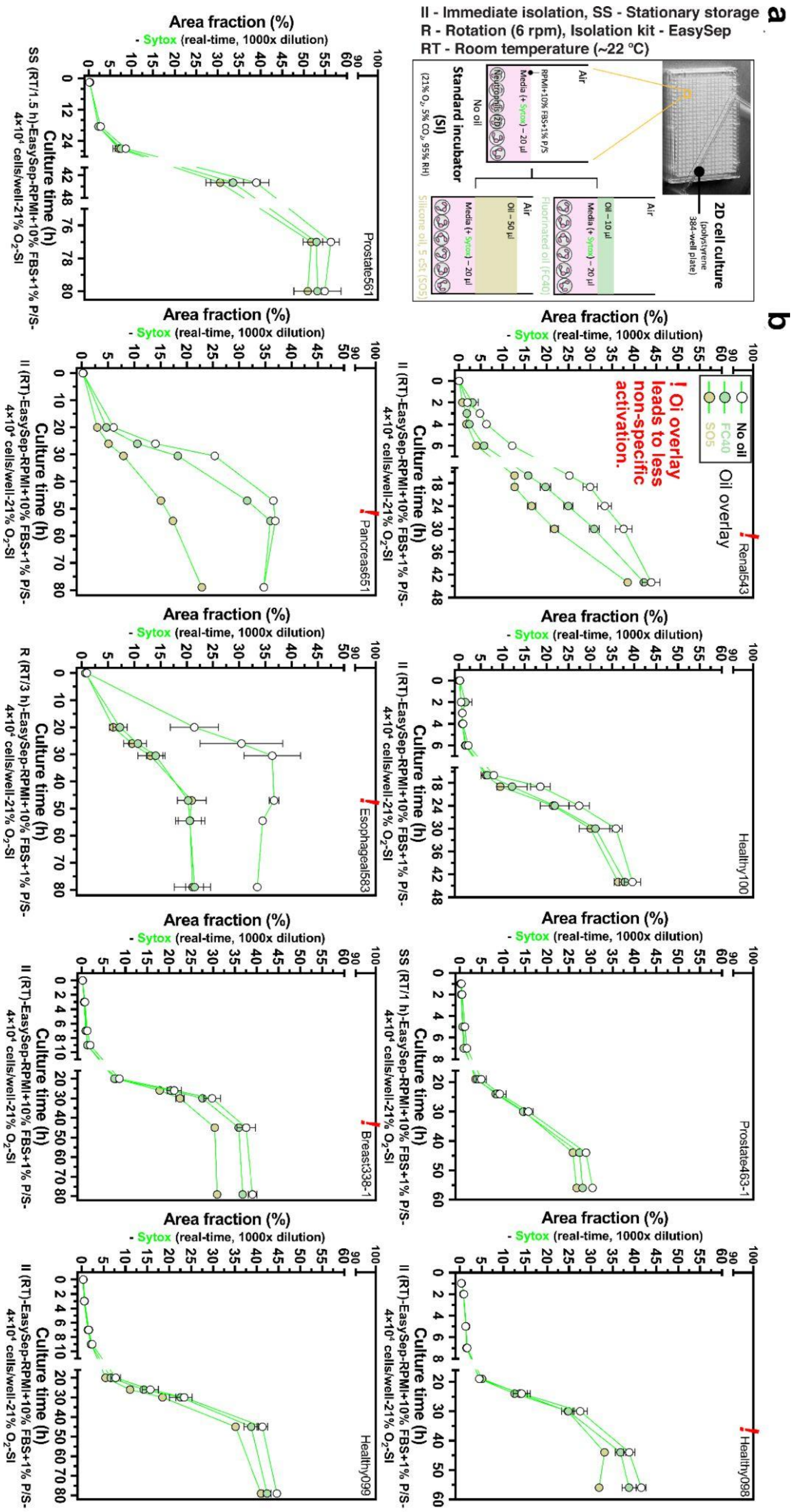
a
b
II - Immediate isolation, SS - Stationary storage
R - Rotation (6 rpm), Isolation kit - EasySep
RT - Room temperature (~22 °C)
2D cell culture
Standard incubator
No oil
Oil overlay
! Oi overlay leads to less non-specific activation.
Area fraction (%)
- Sytox (real-time, 1000x dilution)
Culture time (h)
Renal543
Healthy100
Prostate463-1
Healthy098
Prostate561
Pancreas651
Esophageal583
Breast338-1
Healthy099

Supplementary Fig. 7 Influence of oil overlay on non-specific activation and neutrophil kinetics in standard 2D monoculture. a Schematic showing the culture condition with and without oil overlay in a 384-well plate. **b** Non-specific activation and neutrophil kinetics in culture from nine donors. Results are area fraction from the Sytox (real-time, 1000× dilution) channel. The red "*!*" indicates the experiments where oil (espeically silicone oil) overlay led to significantly less non-specific activations of neutrophils compared to the no oil control.

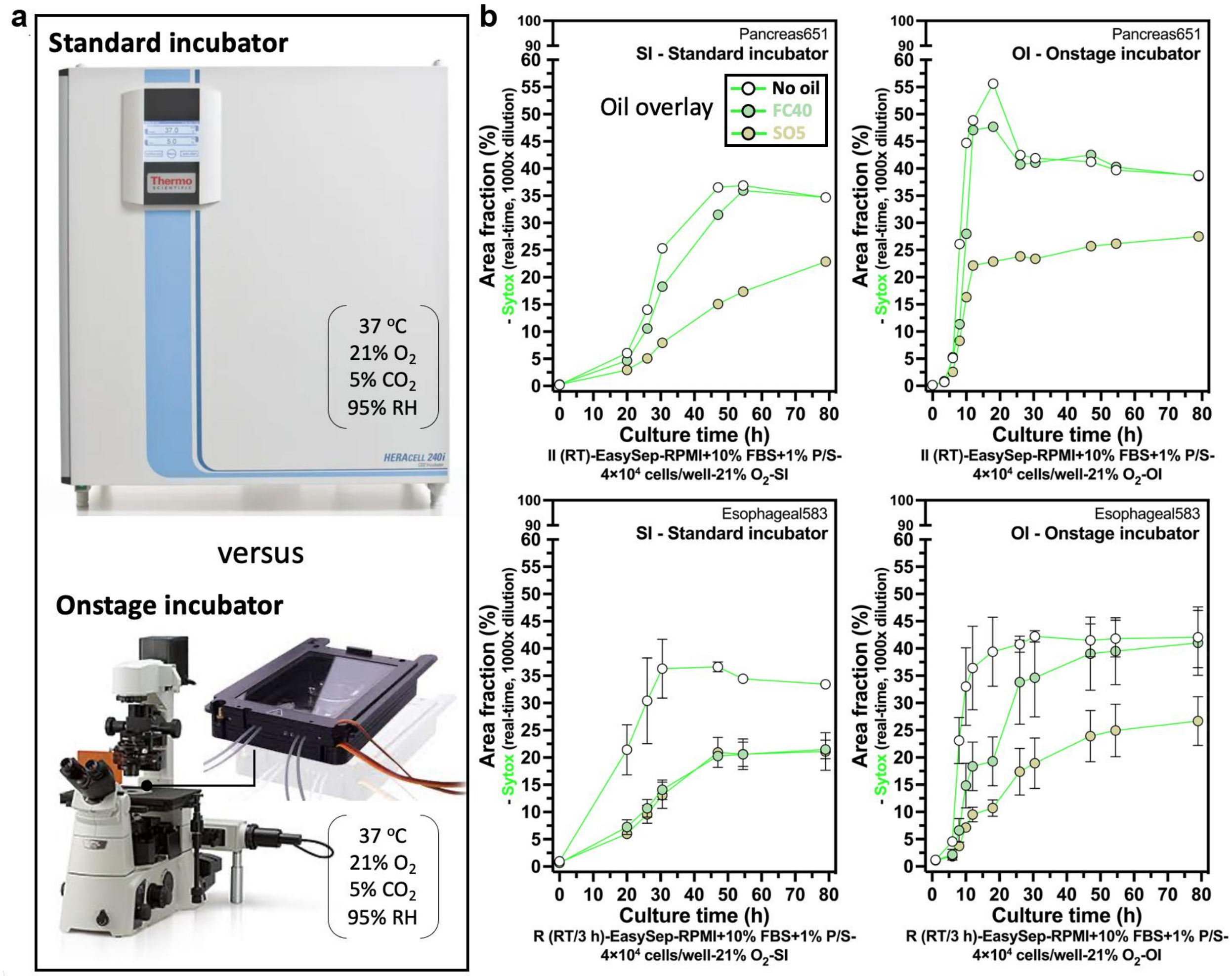

Supplementary Fig. 8 Influence of the type of incubator (standard incubator versus onstage incubator) on non-specific activation and neutrophil kinetics in standard 2D monoculture. a Pictures of the two incubator systems. **b** Comparison between the two incubator systems on non-specific activation and neutrophil kinetics in culture. Results are area fraction from the Sytox (real-time, 1000× dilution) channel. Data from Pancreas651 were extracted from one replicate of each condition due to the limited neutrophil yield given the conditions tested.

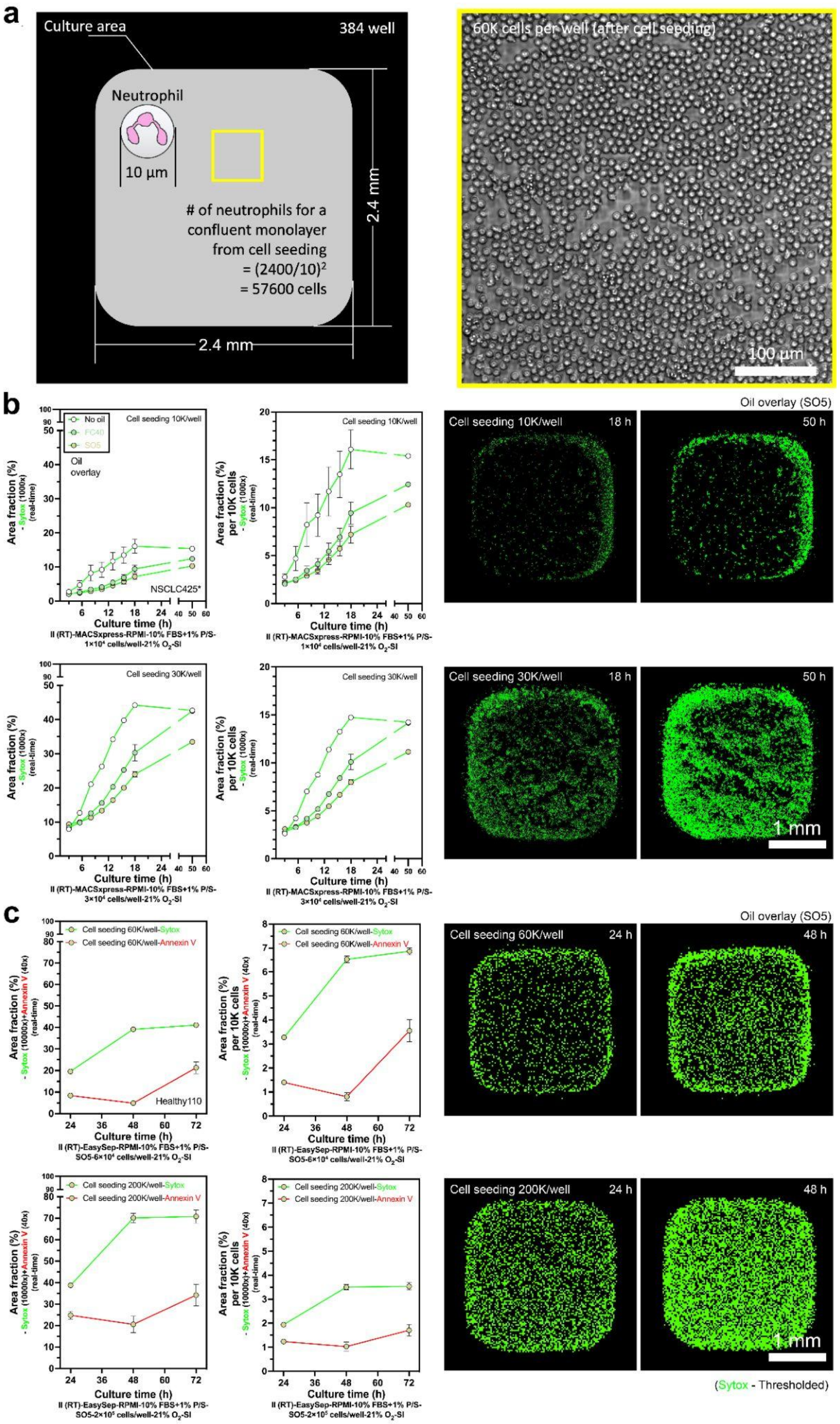
a
Culture area
384 well
Neutrophil
10 µm
# of neutrophils for a confluent monolayer from cell seeding = (2400/10)2 = 57600 cells
2.4 mm
2.4 mm
60K cells per well (after cell seeding)
100 µm
b
No oil
FC40
SO5
Cell seeding 10K/well
Oil overlay
NSCLC425*
Area fraction (%) - Sytox (1000x) (real-time)
Area fraction (%) per 10K cells - Sytox (1000x) (real-time)
Culture time (h)
II (RT)-MACSxpress-RPMI-10% FBS+1% P/S-1×10⁴ cells/well-21% O₂-SI
Oil overlay (SO5)
Cell seeding 10K/well
18 h
50 h
Cell seeding 30K/well
II (RT)-MACSxpress-RPMI-10% FBS+1% P/S-3×10⁴ cells/well-21% O₂-SI
1 mm
c
Cell seeding 60K/well-Sytox
Cell seeding 60K/well-Annexin V
Area fraction (%) - Sytox (10000x)+Annexin V (40x) (real-time)
Area fraction (%) per 10K cells - Sytox (10000x)+Annexin V (40x) (real-time)
Healthy110
Culture time (h)
II (RT)-EasySep-RPMI-10% FBS+1% P/S-SO5-6×10⁴ cells/well-21% O₂-SI
Oil overlay (SO5)
Cell seeding 60K/well
24 h
48 h
Cell seeding 200K/well-Sytox
Cell seeding 200K/well-Annexin V
II (RT)-EasySep-RPMI-10% FBS+1% P/S-SO5-2×10⁵ cells/well-21% O₂-SI
Cell seeding 200K/well
1 mm
(Sytox - Thresholded)

Supplementary Fig. 9 Influence of cell seeding density on non-specific activation and neutrophil kinetics in standard 2D monoculture. **a** Dimension of wells on a 384-well plate and estimation of cell seeding density that leads to a confluent monolayer. **b** 10K versus 30K cells per well. Results are area fraction from the Sytox (real-time, 1000× dilution) with the representative microscopic images. **c** 60K (confluent monolayer from cell seeding) versus 200K cells per well. Results are area fraction from the Sytox (real-time, 10000× dilution) and Annexin V (real-time, 40× dilution) channel with the representative microscopic images.

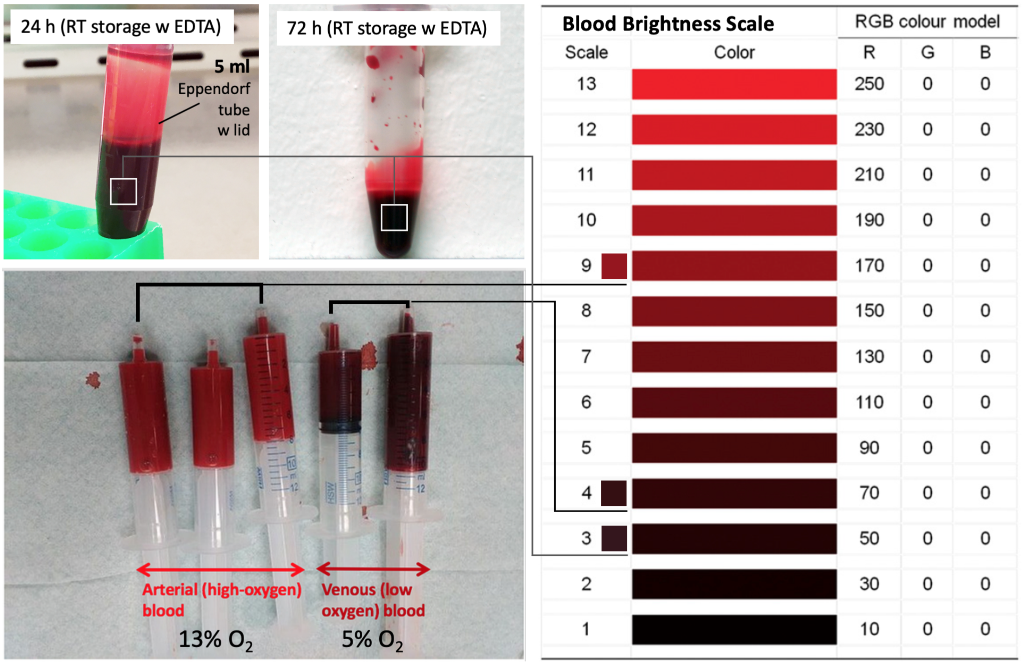

Supplementary Fig. 10 Oxygen level of whole blood (arterial blood, ~13% O_2 versus venous blood, ~5% O_2) and the color of whole blood in storage.
